# Nanosystems for the Encapsulation of Natural Products: The Case of Chitosan Biopolymer as a Matrix

**DOI:** 10.3390/pharmaceutics12070669

**Published:** 2020-07-16

**Authors:** Anastasia Detsi, Eleni Kavetsou, Ioanna Kostopoulou, Ioanna Pitterou, Antonella Rozaria Nefeli Pontillo, Andromachi Tzani, Paris Christodoulou, Aristeia Siliachli, Panagiotis Zoumpoulakis

**Affiliations:** 1 Department of Chemical Sciences, Laboratory of Organic Chemistry, School of Chemical Engineering, National Technical University of Athens, Heroon Polytechniou 9, Zografou Campus, 15780 Athens, Greece; ekavetsou@central.ntua.gr (E.K.); ioanna.th.kostopoulou@gmail.com (I.K.); ipitterou@gmail.com (I.P.); nefelipontillo@gmail.com (A.R.N.P.); andromachi.tzani@gmail.com (A.T.); 2 Institute of Chemical Biology, National Hellenic Research Foundation, Vassileos Constantinou Ave. 48, 116 35 Athens, Greece; parischrchr@gmail.com (P.C.); aristeia.sil@gmail.com (A.S.); 3 Department of Biochemistry and Biotechnology, University of Thessaly, Viopolis, 41500 Larissa, Greece; 4 Department of Food Science and Technology, Universisty of West Attica, Ag. Spyridonos Str., Egaleo, 12243 Athens, Greece

**Keywords:** chitosan, modified chitosan, natural products, encapsulation, nanocarriers, phytochemicals, essential oils, plant extracts

## Abstract

Chitosan is a cationic natural polysaccharide, which has emerged as an increasingly interesting biomaterialover the past few years. It constitutes a novel perspective in drug delivery systems and nanocarriers’ formulations due to its beneficial properties, including biocompatibility, biodegradability and low toxicity. The potentiality of chemical or enzymatic modifications of the biopolymer, as well as its complementary use with other polymers, further attract the scientific community, offering improved and combined properties in the final materials. As a result, chitosan has been extensively used as a matrix for the encapsulation of several valuable compounds. In this review article, the advantageous character of chitosan as a matrix for nanosystemsis presented, focusing on the encapsulation of natural products. A five-year literature review is attempted covering the use of chitosan and modified chitosan as matrices and coatings for the encapsulation of natural extracts, essential oils or pure naturally occurring bioactive compounds are discussed.

## 1. Introduction

Natural products represent a large family of diverse chemical entities produced naturally by any organism with a wide variety of biological activities and distinctive pharmacological effects. They originate from bacterial, fungal, plant, and marine animal sources [1]. They have a wide variety of applications in different sectors such as food, agricultural [2], pharmaceutical [3], packaging [4] application and cosmetics [5] and are often used as flavorings, beverages, repellents, fragrances as well as for their medicinal purposes [6]. Isolated natural products as pure compounds, plant extracts or essential oils have all been used for various applications over the years.

Combining different fields’ approaches including nanotechnology, the optimization of natural products’ features and their wider use is more feasible than ever. This review article will present examples of this combination from well-documented literature.

Encapsulation is a technique in which active agents are entrapped into a biodegradable matrix or “wall” material, forming micro/nano-systems. Encapsulation of bioactive natural compounds, is widely usedin the food, agricultural, pharmaceutical and cosmetic industries and has proved to be a very useful method for: (1) the protection of unstable bioactive compounds from harsh processing conditions (e.g., high temperature, oxygen); (2) the protection of volatile compounds such as essential oils; (3) the construction of targeted delivery systems and controlled-release of the encapsulated compound; (4) the easier handling due to changed physical characteristics of the original core material(change a liquid into a solid); (5) the masking of the undesirable flavors or smells of certain active compounds improving their acceptance as products; (6) the increase in the aqueous solubility etc. [7,8].

Numerous methods for the encapsulation of natural products, such as extracts, essential oils or pure natural bioactive compounds, using various matrices, have been developed over recent years. [8,9,10,11].

Nanoencapsulation can be succeeded via two main approaches: the bottom-up and the top-down. Top-down methodologies include emulsification and emulsification–solvent evaporation while the bottom-up methodologies involve supercritical fluid techniques, inclusion complexation, coacervation, and nanoprecipitation. However, a combination of both approaches is often used. Nanoencapsulation techniques have been used for the encapsulation of both hydrophilic and lipophilic bioactive compounds [10,11].

Microencapsulation (1 μm to 1 mm) offers the potential to deliver active ingredients to desired targets. It could be divided into chemical, physico-chemical and physico-mechanical techniques [12], which include among others spray drying and spray congealing, emulsification, fluid bed coating, ionic gelation, coacervation, centrifugal extrusion, melt extrusion, pan coating method, emulsion solvent evaporation, polymerization and liposome entrapment. Emulsification, coacervation, and supercritical fluid techniques were used for both hydrophilic and lipophilic compounds. Inclusion complexation, emulsification–solvent evaporation, and nanoprecipitation have been used mainly for lipophilic compounds [10,11].

Although a variety of techniques has emerged, no single process is adaptable to all core materials or product applications. Many factors have to be considered before choosing the suitable method [9].

Among all the encapsulation techniques, spray-drying, ionic gelation, emulsification and coacervation (simple or complex) methods are the most widely used [12].

Spray-drying is a simple, rapid and economical, commercial process in which the bioactive compounds are usually homogenized with the matrix and then the mixture is fed into a spray dryer and atomized with a nozzle or spinning wheel. Water is evaporated by the hot air contacting the atomized material and then the capsules fall to the bottom of the drier and are collected [13]. Additional advantages, mentioned in the extended review conducted in 2019 by Assadpour and co-workers [14], are the ability of the liquid feeds to turn into powder form, a property particularly useful for food applications, the higher stability, possibility of large-scale production in continuous mode, lower storage and transport costs, and easier usage.

Ionic gelation is a mild, simple and organic solvent-free approach for the formation of stable nanoparticles. This approach is based on the interaction between oppositely charged macromolecules and a nontoxic and multivalent material in order to provide the charge density. The high loading capacity is considered as an advantage; however, the large particle sizes, pH sensitivity, and high polydispersity are the main drawbacks of this method [2,3,4].

Emulsification process is based on heterogeneous structures comprised of two liquid phases (water-in-oil, W/O, oil-in-water O/W), dispersing one liquid in another as droplets and stabilized by an appropriate emulsifier [15]. Emulsions are unstable and this is the reason why surfactants are incorporated in the system. O/W emulsions are commonly used for essential oils (EOs), where oil droplets are spread in a water-medium and stabilized with food-grade surfactants or biopolymers.

Recently, Kumar et al. [15] reviewed the encapsulation of bioactive compounds using nanoemulsions targeting in food processing applications. The coacervation procedure is one of the oldest and most widely used encapsulation techniques in the food, cosmetic and pesticides industries [16]. Depending on the number of polymers involved, it can be classified as a simple or a complex technique, yet the latter is preferred by the food and pharmaceutical fields. This method is based on the separation of two liquid phases in a colloidal solution. It is used to encapsulate heat-sensitive ingredients but it is an expensive procedure, requiring the use of toxic agents while the complex coacervates are unstable [10].

Choosing the suitable matrix material for the intended application is of great importance, since it affects the encapsulation efficiency (EE) and the stability of the formed nanostructuresas well as the release profile of the encapsulated molecule [9]. Natural macromolecules such as polysaccharides, oligosaccharides, proteins, synthetic polymers and lipids have been applied as matrices for the encapsulation of a variety of natural and synthetic molecules.

Cyclodextrins (CDs) are natural oligomers widely used for the encapsulation of natural products. They are derived from starch and they are composed of several D-glucose units. The arrangement of the monomers can be simulated with a truncated cone, where the secondary and primary hydroxyl groups are projected on the wide and narrow rim, respectively, forming a hydrophilic exterior, while CH_2_ groups and glucosidic oxygen atoms consist the hydrophobic interior cavity of the cone [17,18].

Scientists all over the world have thoroughly examined and characterized the entrapment of pure natural compounds (e.g., polyphenols [19,20], flavonoids [21,22], alkaloids [23,24,25], terpenoids [26]), EOs [27,28] (e.g., tee tree oil [29], oregano [30], *Eucalyptus staigeriana* [31]) and extracts (e.g., propolis [32,33], olive leaves [34], *Chimonanthus praecox* extract [35]) in α-, β- or γ-CDs or modified CDs for several applications, revealing significantly improved properties to the final complexes [36].

Chitosan is a unique cationic polysaccharide, with well-known antioxidant, lipid-lowering and antimicrobial activities, film-forming and gelling properties, encapsulation potential, etc. This biopolymer is considered as GRAS (Generally Recognized as Safe) by the Food and Drug Administration (FDA). Numerous are the potential applications of chitosan in different fields, such as food, pharmaceutical and cosmetic [37]. The cationic nature of chitosan leads, under acidic conditions, to the development of various forms, such as nano/micro-particles, emulsions, fibers, hydrogels, films and membranes. Chitosan in its various forms, has extensively been used as a matrix for the encapsulation of extracts, EOs and bioactive compounds.

Alginate is a commonly used encapsulation matrix for a variety of materials such as plant or mammalian cells, yeasts, bacteria and food products, drugs, oils and flavors as it forms stable reversible gels, it is cheap and available. It is capable of absorbing water quickly (taking 200–300 times their own weight in water) forming a kind of a viscous gum [38]. The combined use of calcium alginate with other biomaterials such as chitosan [39] could lead to a new way of controlling drug delivery.

Hydrocolloids or, more commonly, gums are aclass of wall materials often exploited for their encapsulating abilities [40]. Gum arabic or gum acacia is a natural gum, collected from *Acacia arabica* wild. It constitutes an acidic anionic polysaccharide which isfrequently used by various industries due to its highly emulsifying and good encapsulating properties, high solubility and low viscosity [41]. Gums have been used for the encapsulation of EOs (e.g., peppermint [40], citronella [42] and pure bioactive compounds [43]) but most commonly they are used in combination with other natural polymers [44,45,46].

Carrageenan is a high molecular weight, negatively charged, water-soluble sulfated polysaccharide isolated from red algae. Carrageenan has been used as a gelling agent, control release vehicle, and encapsulating agent. The carrageenan gels, beads and films, can efficiently encapsulate flavors, fragrances, probiotics, and enzymes [47]. Recently carrageenan has been used for microencapsulation of caffeine [48] and also EOs such as pimento [49], cardamom [50] along withother polysaccharides (e.g., chitosan, gums) and proteins (e.g., gelatin, whey protein) mainly using the complex coacervation method.

Starch is one of the most abundant and low-cost natural polymers, it is generally recognized as GRAS and has recently used in microencapsulation of extracts such as *Hibiscus sabdariffa* [51] anthocyanin [52] extract. Its combined use with other natural polymers for encapsulation processes has also been investigated [53,54,55].

## 2. Chitosan as a Matrix for NanosystemsPreparation: Methods, Physicochemical Aspects, Modification Potential and Bioactivity

Chitosan has gained attention due to its beneficial properties and the vast variety of its potential applications. Numerous studies have been conducted describing the different properties of the polymer. Chitosan is derived from the deacetylation of chitin, a naturally occurring polymer, and is known for its biocompatibility, biodegradability, and low toxicity. One of the most attractive properties of chitosan is the ability to form nanostructured formulations viarapid and mild procedures. Akbari-Alavijeh and co-workers [56], in their recent review, categorize the different nano-carriers prepared with chitosan in nanoparticles, nanofibers, nanogels, nanocomposites and nanocoatings.

Chitosan nanoparticles (CSNPs) are prepared using several methods. The most commonly used for the encapsulation of natural products are ionic gelation, spray-drying and emulsification [56].

**Figure 1:**
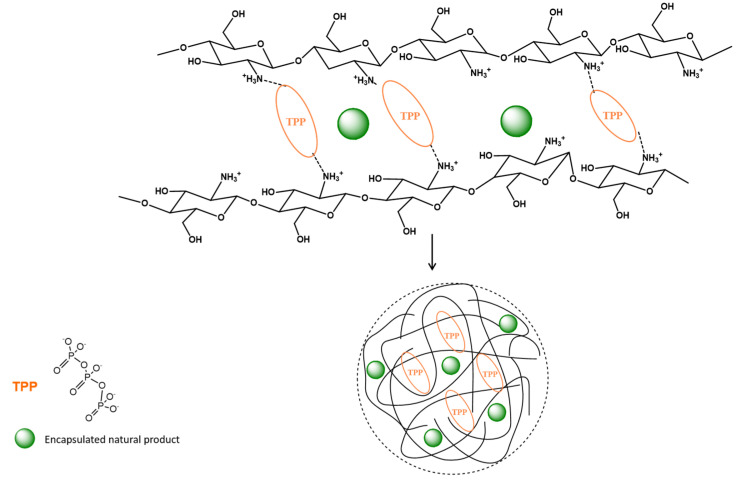
Schematic representation of the formation of chitosan nanoparticles encapsulating natural products via the ionic gelation technique.

Regarding ionic gelation, it occurs due to the inter- and intra-molecular cross-linking of the polycationic chitosan by an anionic cross-linker such as the most commonly used tripolyphosphate (TPP) as depicted in Figure 1. In this method, chitosan is dissolved in acetic acid aqueous solution, and the aqueous solution of TPP is then added dropwise into the chitosan solution. Nanoparticles are formed instantly under mechanical stirring at room temperature [57,58,59].

In thespray-drying method, chitosan is dissolved in an aqueous solution of acetic acid, the compounds to be encapsulated are suspended or dissolved in the solution and then a selected cross-linking agent (such as TPP [60] or d,l-glyceraldehyde [61]) is added [58]. This solution is then atomized into a chamber.

The emulsification–solvent diffusion technique is based on the crosslinking between the reactive functional amine groups of chitosan and aldehydes (such as glutaraldehyde, formaldehyde) or even vanillin as an eco-friendlier alternative. Chitosan precipitation occurs upon the diffusion of organic solvent into water [58].

**Figure 2:**
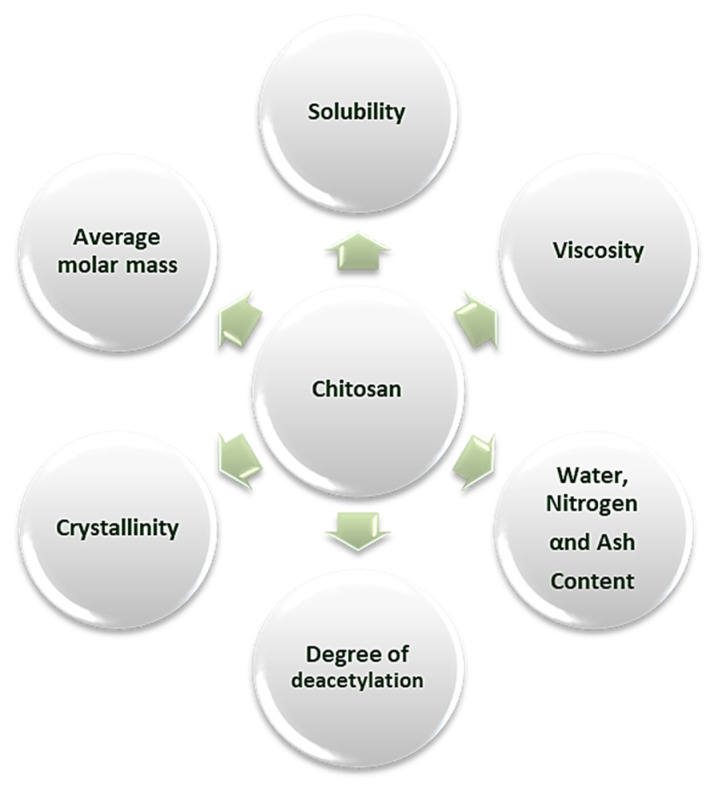
Physicochemical Properties of Chitosan.

The physicochemical properties of chitosan define the relationship that exists between the chemical structure of chitosan and its uses in many branches of science and industry (Figure 2). Deacetylation degree (DD), average molar mass, solubility, crystallinity, viscosity and content of water are the most frequently evaluated properties [61,62,63].

The physicochemical properties of chitosan derived from various sources such as shrimp, fish and crab have been evaluated by Kumari et al. [64]. Briefly, shrimp shells have proven to be the best choice for the preparation of chitosan and this was confirmed by all the physicochemical properties such as high average molecular weight (MW), high DD value and solubility data.

Oh et al. [65] evaluated the physicochemical characteristics of chitosan-based film forming gel containing ketoprofen (CbFG). More specifically, CbFG was prepared with chitosan, lactic acid and various skin permeation enhancers. In this study, CbFG containing oleic acid had a higher skin permeation rate in comparison with any other candidate enhancers. Consequently, the grafting of chitosan onto oleic acid performed a key role in the inhibition of crystal formation of ketoprofen in the CbFGfilms [66].

L. de Souza Soares et al. [67] studied the dispersibility of chitosan in aqueous solutions containing lactic acid, acetic acid, glycolic acid or propionic acid at different concentrations (10, 20, 30, 40, or 50 mmol·L^−1^). The increase in acid concentration reduced pH and viscosity of the dispersions, andzetapotential of the dispersed particles. In contrast, it increased the values of electrical conductivity, density, and hydrodynamic diameter of the dispersed chitosan particles. All of these effects were slightly more pronounced for dispersions formed with glycolic or lactic acids, compared to acetic or propionic acid. Chitosan chains interacted more strongly with hydroxylated acids counter-anions (glycolate and lactate) than with their non-hydroxylated counterparts (acetate and propionate), leading to slight quantitative changes of physicochemical properties of these systems. Consequently, in physicochemical terms, glycolic, lactic or propionic acids are suitable to replace acetic acid when preparing aqueous chitosan dispersions for technological objectives. 

Hong et al. [68] studied the physicochemical properties of modified chitosan. More specifically, chitosan was modified using H_2_O_2_ and ascorbic acid under different incubation temperatures. Modified chitosan increased water solubility at pH 7 and showed improved viscosity properties compared to non-modified chitosan. Additionally, modified chitosan worked effectively as a lipid digestion inhibitor. 

Furthermore, Panda et al. [69] modified chitosan of three different MWs by using p-coumaric acid for enhancing their water solubility and antioxidant properties. The results showed that the water solubility and antioxidant properties of the modified product decrease, when the MW of corresponding native chitosan increases. However, modified product had good solubility over a wide range of pH.

Cheng et al. [70] reported physicochemical properties of chitosan prepared by microwave and water bath heating with an equivalent quantity of heat intake. Briefly, chitosan production with microwave heating reduced the time of deacetylation from 180 to 60 min to reach the same DD% as the water bath heating with the same quantity of heat. Moreover, the chitosan produced by microwaves can obtain relatively low MW and viscosity. These results showed that microwave was a more efficient, energy-saving, and environmentally friendly method for the further use of rigid shrimp shells and highly crystalline crustacean materials.

Apart from CSNPs, other nanosystems based on the chitosan matrix contribute to the wide array of advantages of this natural polymer such as nanofibers, nanogels, nanocomposites and nanocoatings. Nanofibers (1 to 1000 nm) are solid fibers characterized by superior porosity and the most commonly used preparation method is the electrospinning method. The low solubility, rigid structure and high crystallinity nature of chitosan lead to low electrospinability and this is the reason why chitosan is used as a mixture with compounds such as polylactic acid, trifluoroacetic acid etc [57]. Nanogels are nano-sized hydrogels and the cross-linking method is the most commonly used for their preparation. Chitosan can form hydrogels with no additives due to its polycationic nature and the charge density of hydrogels could be controlled by modification of the ionic strength and pH. The chitosan-based hydrogels are a promising matrix for tissue engineering and regenerative medicine [71,72].

Finally, the chitosan nanocomposites and nanocoatings are mainly obtained by dispersing of nanoscale filler into chitosan which is a natural polymer. The two main strategies to obtain nanostructures and, consequently, polymer nanocomposites and nanocoatings are the bottom-up and bottom down methods. The first one is an in-situ synthesis method (e.g., in situpolymerization, spin coating, casting) while the second is anex situsynthesis (attachment of nanoparticles to the polymer matrices prepared into different steps) [73]. 

All different chitosan formulations, can be used as carriers of valuable compounds, protecting them from degradation or even adding or even altering some of their properties, such as enhancing their poor solubility in water and improving their thermal stability [3]. The high surface to volume ratio of nanoparticles permits the controlled and sustained release of the encapsulated compound, allowing the design of a targeted mode of action and delivery system.

The cationic nature of chitosan attributed to the presence of amino groups which are protonated in acidic environment, enables the electrostatic interaction with the anionic mucin in comparison with other anionic biocarriers (e.g., β-CD and alginate). The mucoadhesive property of the polymer, is an asset for non-parenteral [74,75,76] drug delivery, prolonging the contact time and, thus, the absorption time and bioavailability of the bioactive compound. Moreover, the physical defence of the body which leads to inflammation has a positive impact on the targeted action of chitosan’s nanoparticles. The acidic environment and the slightly increased temperature of an infected area, results in higher dissolution of chitosan, therefore, increased drug release. 

The antimicrobial [77,78], antioxidant [79] and lipid-lowering activities of chitosan, are enhanced in the nanoparticulate form. This could be attributed to the high surface to volume ratio, reinforcing the properties of the encapsulated compounds or natural products. Krausz et al. [80] report the enhancement of the antimicrobial and wound healing activity of encapsulated curcumin, while the research of Kavaz et al. [81] showed that the encapsulation of *Cyperus articulates* EO enhanced the antibacterial activity against *Staphylococcus aureus* (ATCC6538) and *Escherichia coli* (ATCC 8739) and the in vitro cytotoxicity against MDA-MB-231 breast cancer cells. Taher et al. [82], verified the higher in vitroanti-proliferative effect of chitosan nanoparticles against two different human breast cancer lines compared to microparticles. 

Chitosan modification through chemical or enzymatic methods is an additional asset and a very promising tool for the development of new nanosystems. The existence of three functional groups—the primary amine and the primary and secondary hydroxyl groups—allows the modification of the polymer which could alter its physicochemical properties but maintaining or enhancing its core properties. One of the most common goals is the increase in the polymer’s solubility, the change of the surface charge or the modification of the release profile of the encapsulated compound [83].

**Figure 3:**
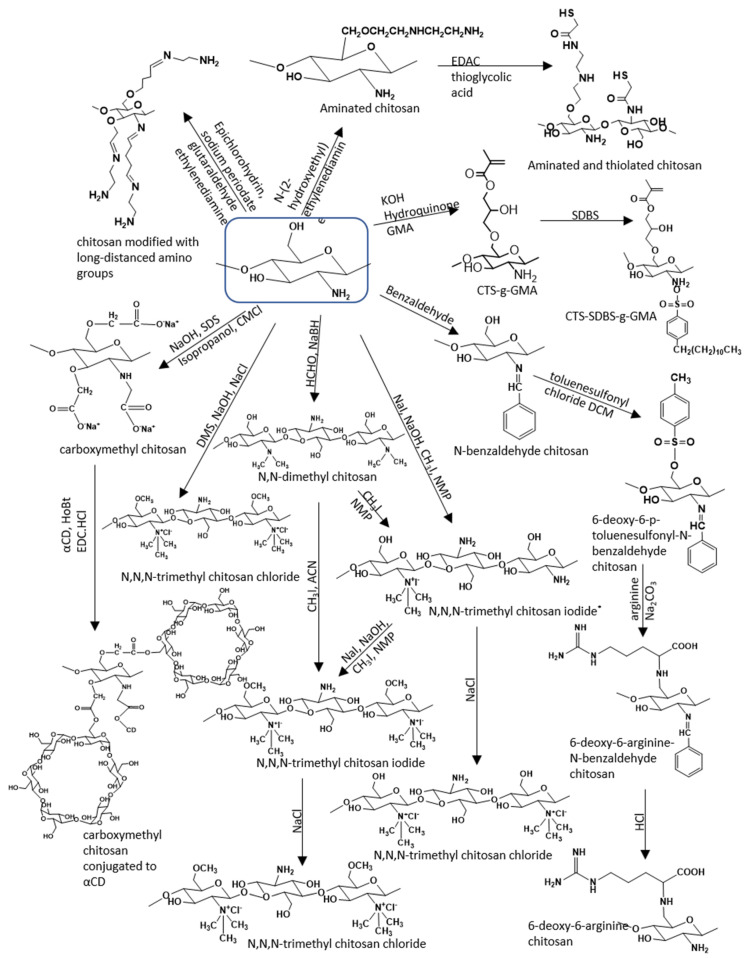
Schematic illustration of the synthesis of different chitosan derivatives [83,84,85,86,87,88].

The modification of the primary amino group is the most common as it can undergo many different reactions like methylation, acylation, thiolation, Schiff base reaction, graft copolymerization (Figure 3). A chemically modified chitosan derivative that is highly investigated is the *N, N, N*-trimethyl chitosan, as it is more soluble in water and possesses mucoadhesive properties and can also be used as a nanocarrier [84]. The Schiff base reaction is also often employed for the synthesis of more complex chitosan structures. Su et al. [84] synthesized the *N*-benzaldehyde chitosan which was further modified to form the non-toxic, water soluble 6-deoxy-6-arginine- modified chitosan that exhibits high antibacterial activity. 

The primary amino group can be modified via reactions like elimination/condensation reaction and amidation. Sinani et al. [85] modified both the primary amino and the primary hydroxyl group to synthesize an aminated and thiolated chitosan that could be used as a nanoparticulate system with enhanced mucoadhesive properties. García-Valdez et al. [86] functionalized the primary hydroxyl group with glycidyl methacrylate (GMA) and then the primary amino group with sodium dodecylbenzene sulfonate (SDBS), and used the complex for the synthesis of polystyrene and poly(*n*-butyl acrylate) modified chitosan. Chitosan modified with long-distanced amino groups is selected for the coating of magnetic nanoparticles designed for biomedical applications [87] because it ensures the extension of the physicochemical properties.

The complementary use of chitosan with other polymers, such as alginate, hyaluronan, poly (lactic-*co*-glycolic acid) (PLGA), oligosaccharides like CDs or macromolecules as corn zein and quinoa proteins is also very promising.The above-mentionedcombinations can lead to advanced properties, tailored according to the end-use application. Saekhor et al. [88], conjugated carboxymethyl chitosan with α-CD. The solubility of chitosan increased and an injectable hydrogel that could be used as a bone tissue engineering scaffold was prepared. 

Chitosan could be employed in a vast variety of applications. The aforementioned properties, along with the ability of this biopolymer to be transformed to various forms, such as nano/micro-particles, emulsions, fibers, hydrogels, films and membranes are the reasons why chitosan has gained so much attention as a valuable polymer [37]. In biomedicine, chitosan could be used for treatment or diagnosis of cancer, rheumatoid arthritis, diabetes or in wound dressing applications [89,90,91] etc. In agriculture, chitosan nanoparticles were found to have a positive impact on the growth of fish and wheat [92,93].

Chitosan biopolymer has been extensively used as a matrix for the encapsulation of a wide range of natural products. In this review focus is given to the last five years, which have been very productive regarding the preparation of chitosan nanoparticles loaded with pure bioactive naturally occurring compounds, EOs or extracts. 

Over the years, various natural bioactive ingredients such as tea polyphenols [94,95], curcumin [96], flavonoids (e.g., genistein [97], baicalein and quercetin [98], kaempferol [99]), phenolic compounds [100] (e.g., ferulic acid [101]) have been encapsulated in chitosan nanoparticles.

As far as EOs are concerned, an extensive review of the literature shows that, over time, they have been increasingly attracting the interest of researchers. Clove [102,103,104,105,106] and thyme [107,108,109,110,111] EOs have been largely used in encapsulation studies, mostly through the ionic gelation technique, targetingdifferent applications. However, a wide range of EOs-based chitosan nanoparticles have also been referred to the literature over the last five years, such as mint (*Mentha piperita*) [111,112], cardamom [113], krill oil (*Euphausia superba*) [114], lime [115], orange [116], lavender [109,116], *Achillea millefolium* [117], *Cymbopogon martini* [118], citrus [119], *Piper nigrum* [120], peppermint or green tea EOs [3,121].

Natural extracts are a widely investigated class of natural products, often implemented for the formation of chitosan particles. Over the past five years, several research teams have presented the synthesis of chitosan micro- or nano-particles loaded with a plethora of extracts, such as *Centella asiatica* [122], *Cinnamomum cassia* [123], *Plinia cauliflora* (jabuticaba) fruit peel [124], *Origanum vulgare* L [125], *Mentha longifolia* [126], *Physalis alkekengi*-L [127], *Eugenia dysenterica* [128] or grape and apple pomace phenolic extract [129], aiming at their enhanced characteristics in order to be applied in various fields.

## 3. Chitosan as a Matrix for the Encapsulation of Pure Phytochemicals 

Phytochemicals are a wide variety of chemical compounds occurring naturally in plants. They are associated with health benefits [130,131] but are not considered as essential. Phytochemicals are classified into six major categories containing many subcategories: carbohydrates (monosaccharide, disaccharide, polysaccharide, oligosaccharide, sugar alcohols), lipids (monounsaturated fat, polyunsaturated fat, saturated fat and fatty acids), polyphenols (A:phenolic acids: A1:hydroxybenzoic acids and A2:hydroxycinnamic acids, B:flavonoids, B1:flavones, B2:flavonols, B3:flavan-3-ols, B4:isoflavones, B5:flavanones, B6:anthocyanidins and anthocyanins, C:other phenolics, C1:stilbenes, C2:lignans, C3:tannins, C4: xanthones, C5:lignins, C6:chromones, C7:anthraqyinones), [132] terpenoids (mono-, di-, tri-, sesqui-terpene and carotenoids) alkaloids, and other nitrogen-containing compounds (aliphatic mono- and poly-amines, amino acids, proteins) [130,131] and cannabinoids.

Many pure phytochemicals have been extensively studied and their biological properties, pharmacokinetic profile and desirable therapeutic effect are well defined, facilitating the dose determination. In terms of safety, as with drugs, a single compound could interact withfewer receptors in the human organism than the mixture of compounds in an extract, thus, an isolated and purified natural product is considered as safer when administered in concentrations found in nature. Furthermore, when natural products are used in agriculture, they have many advantages, such as low persistence in the field, strong selectivity and complexity that can delay the development of resistance in target organisms [133].

Despite isolated phytochemicals’ safety, effectiveness and multiple pharmacological properties, their use is limited because of their low bioavailability, owing to their rapid elimination, poor adsorption [134] and stability. Often phytochemicals with excellent in vitro biological activities and efficacy ratio failed to replicate these results in in vivo animal models and clinical trials [135]. Indeed, for some categories, such as polyphenols, only a small quantity after ingestion is absorbed from the gastrointestinal tract and reaches blood circulation [136]. Some bioactive components can also undergo enzymatic oxidation or degradation in many food processes or storage and form even harmful components. Other phytochemicals, such as catechin, are unstable in gastrointestinal solutions [137]. Poor aqueous solubility of most phytochemicals is another limitation [135,138,139]. Moreover, the surfactants and/or solubilizing agents used during the extraction of the bioactive compounds may decrease their concentration [135]. The increased dose of plytochemicals required for the desired beneficial therapeutic effect is also posing a strong restriction of their use [140]. This often results in selective or functional toxicological complications due to loss, inactivation and/or degradation during transport of the molecule from the site of administration to the target site [135]. 

It is therefore imperative to look for a carrier system for the pure natural ingredients that can eliminate most of the aforementioned limitations in order to improve the clinical outcome [136]. Nanoencapsulation is an effective strategy to overcome these limitations byenhancing the targetability, prolonging the release rate of the encapsulated product and ameliorating the stability of the substances. For instance, upon ingestion, nanoparticles containing pure phytochemicals could adhere to the mucosa of gastrointestinal tract, due to their characteristic mucoadhesive properties [136], and then be transported via circulation to different organs-targets elongating their therapeutic effect [138]. 

Chitosan is one of the most widely used encapsulating agents and several reports have been published recently especially for polyphenolic compounds [138], most of them using the ionotropic gelation method with TPP (Table 1) [99,101,134,135,138,139,141,142,143,144].

**Figure 4:**
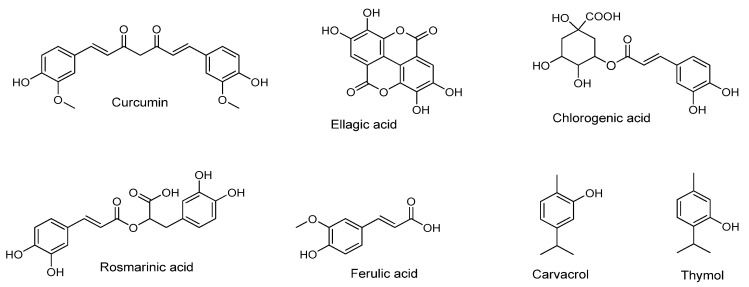
Chemical structures of selected phenolic compounds mentioned throughout this review.

Regarding hydroxycinnamic acids, Nallamuthu et al. [138] studied the encapsulation of chlorogenic acid (Figure 4) in chitosan spherical shaped nanoparticles by ionic gelation method. The EE was determined to be up to 59%, the loading efficiency up to 5% and the size of the particles was 210 nm. The study showed controlled release profile, preserved antioxidant activity and increased bioavailability. Ferulic acid (Figure 4) has been encapsulated in chitosan nanoparticles showing improved pharmacokinetic profile [145] and a range of therapeutic effects [142] such as lowering high glycemic blood levels and avoiding secondary complications associated with the synthetic drugs in rats [146] as well as antifungal activity [101]. Panwara et al. [141] encapsulated ferulic acid in chitosan byionic cross-linking of chitosan with TPP. Smooth and spherical ferulic acid-loaded nanoparticles (NPs) were prepared of an average diameter up to 125 nm which could be exploited as a therapeutic agent against cancer cells proliferation. Da Silva et al. [143] prepared rosmarinic acid-loaded chitosan nanoparticles (200–300 nm) by ionic gelation using TPP at pH 5.8 and they studied their potent use as a promising delivery system for ocular applications in oxidative eye conditions [143].

Ellagic acid (Figure 4), a hydroxy benzoic acid derivative, was encapsulated in chitosan nanoparticles using the ionic gelation method with TPP as the cross-linking agent. The release profile of ellagic acid from the formed nanoparticles at pH 7.4 showed that 84% of the compound was released after 12 h which is a desirable property for the use of the nanosystem as an effective antihemorrhagic agent [134].

Yadav et al. [140] entrapped pure curcumin in chitosan cross-linked with glutaraldehyde. The loaded nanoprticles had a diameter of less than 50 nm and thecalculated entrapment efficiency was >90%. The curcumin-loaded nanoparticles were shown to be a stable detoxifying agent for arsenic poisoning [140]. Akolade et al. [135] used chitosan-alginate complexes to successfully encapsulate curcumin (<50 nm) for reduction of hyperglycemia. The EE was found to be 64 to 76%, the loading capacity 20 to 26% and the yield ranged between 50 and 72%. In the study of Das RK. et al. [147], the curcumin-loaded chitosan-alginate-pluronicnanoparticles (spherical of an average size up to 100 ± 20 nm) were prepared by ionotropic pre-gelation followed by polycationic cross-linking andshowed improved characteristics. Gupta et al. [148] loaded curcumin in chitosan-silk fibroin nano-matrices using the devised capillary-microdot technique. The resulting nanomaterial showed weak efficacy against breast cancer [148]. The nanoparticles were less than 100 nm in size and the EE was found to be 64% and 73%. In the work of Sun et al., curcumin was encapsulated in aminated chitosan, modified with folic acid in order to investigate the ability of this modification to stabilize curcumin and facilitateslow release of the natural product in different pH environments. The nanoparticles comprising of folic-acid-modified aminated chitosan showed targeted cytotoxicity against tumor cells and a cumulative release rate which depended on the pH of the medium (56% in 48 h at pH 7.4 and 89% in 24 h at pH 1.2) [149].

The mucoadhesive properties of chitosan can be exploited in order to synthesize nanoparticles with enhanced activity against colorectal cancer because they can ensure prolonged contact with the colon and sustained release of the encapsulated bioactive compound. Thus, Chuah et al. [150] prepared curcumin-loaded chitosan nanoparticles using the ionic gelation method with TPP and found that the nanoparticles showed better anticancer activity against colorectal cancer and improved cellular uptake compared to free curcumin.

**Figure 5:**
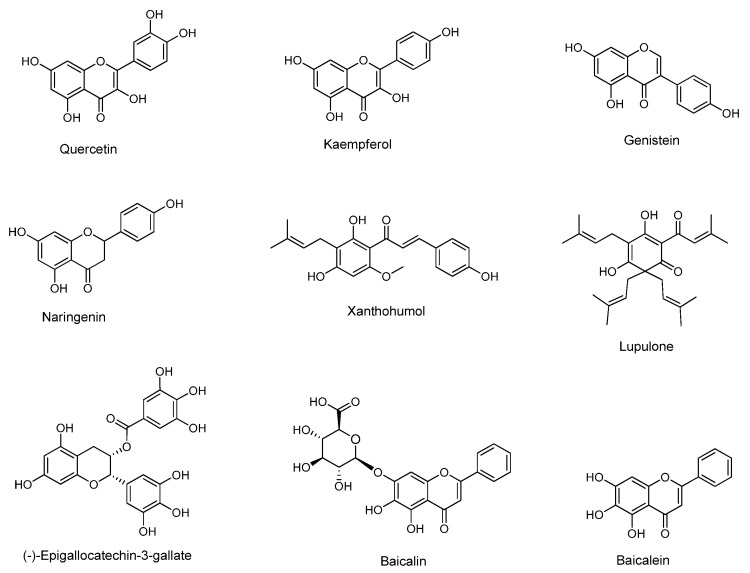
Chemical structures of selected flavonoids mentioned throughout this review.

In the subcategory of flavonols (Figure 5), quercetin-loaded chitosan nanoparticles with improved bioavailability [139] as well as kaempferol-loaded chitosan nanoparticles as a quorum sensing anti-biofilm agent for antimicrobial chemotherapy [99] have been reported. The study by Ilk et al. [99], indicated that the average kaempferol-loaded chitosan/TPP nanoparticle size were 192.2 ± 13.6 nm and the loading and EE of kaempferol into nanoparticles presented values between 78 and 93%. Kumar et al. [142] used the ionic gelation method with TPP to encapsulate the flavanone naringenin into chitosan nanoparticles. The study revealed that the particulate system sized up to 407.5 nm with EE between 70 and 80%, showed significant antioxidant and cytotoxicity against lung cancer cells [142].

The flavonoids quercetin and baicalein (Figure 5) incorporated in chitosan nanocapsules were tested by Omwenga et al. [98] for their ability to inhibit biofilm formation and quorum sensing as well as their cytotoxicity on mammalian cells. The association efficiency was determined up to 99% for quercetin and 87% for baicalein and each formulation had an average diameter of 190 ± 4 nm and 187 ± 2 nm, respectively. Free quercetin and free baicalein were cytotoxic to MDCK-C7 cells but no toxicity was observed by their nanoencapsulated form. Moreover, the flavonoid-loaded chitosan nanoparticles presented enhanced inhibition of biofilm formation and quorum sensing of bioengineered *Escherichia coli* [98].

Baicalin, the glucoronide of baicalein, is a natural compound frequently met in herbs and used in Chinese Traditional Medicine. It has a wide range of bioactivities such as anti-inflammatory, antihypertensive, antifungal, antioxidant, neuroprotective and many more. Nevertheless, in spite of its promising bioactivity profile, baicalin has very low bioavailability and short half-life which do not make it a good candidate drug for development. Saad Ahmed et al. [151] used chitosan lactate, which was further modified with lactobionic acid as a matrix to prepare baicalin-loaded nanoparticles to target the liver. The ionic gelation method with TPP as the cross-linker was used for the preparation of the nanoparticles. The results from thein vivobiodistribution showed that the nanocarrier increases the concentration of baicalin in the liver compared to the free baicalin, indicating that the modified chitosan is efficient for this application [151].

(-)-Epigallocatechin-3-gallate (EGCG) (Figure 5), a flavan-3-ole, encapsulated in chitosan nanoparticles has shown enhanced oral delivery and therapeutic application for many diseases [136,137]. Lupulone and xanthohumol (Figure 5) extracted from the plant *Humulus lupulus* L. wereincorporated in chitosan nanoparticles showed good stability and antimicrobial activity for many applications [144]. Zu et al. [152] entrapped resveratrol in carboxymethyl chitosan. The synthesized spherical nanoparticles possesed average particle size, drug loading and EE of 155.3 ± 15.2 nm, 5.1 ± 0.8% and 44.5 ± 2.2%, respectively, and showed better bioavailability in an in vivo study on rats.

The isoflavonoid phytoestrogen genistein (Figure 5) was encapsulated in CSNPs using ionic gelation methodology with sodium hexametaphosphate, a non-toxic polyanion as an alternative cross-linker. The nanoparticles possessed 200–300 nm mean size and were found to enhance genistein penetration through the nasal mucosa as compared to free genistein while they preserved PC12 cell vitality [153]. Hence, chitosan can be regarded as a promising nanocarrier for intranasal delivery of genistein.

Spherical CSNPs containing the natural phenolic compound eugenol were prepared via the emulsification-ionic gelation methodology. The nanoparticles were incorporated in thermoplastic flour via extrusion and the resulting material showed higher antioxidant activity than the one containing non-encapsulated eugenol indicating its potential use in active packaging applications [154].

**Figure 6:**
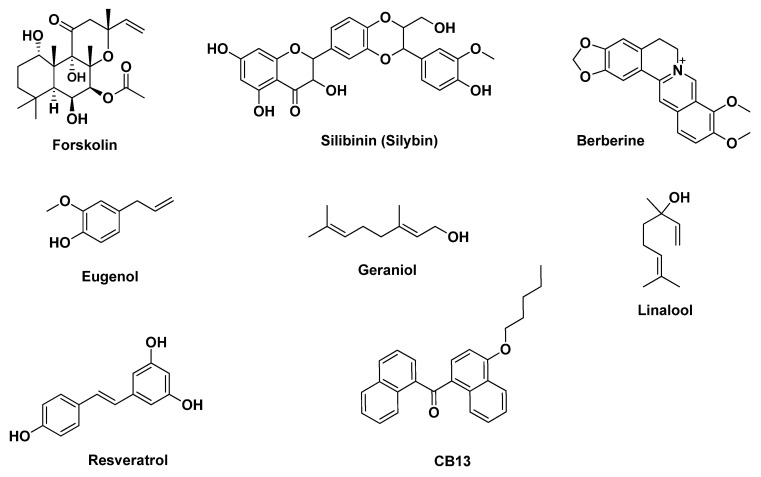
Chemical structures of natural products belonging in various structural families encapsulated in chitosan or modified chitosan nanoparticles (CSNPs).

There are few studies describing pure phytochemicals other than polyphenols encapsulated in chitosan. Such an example is berberine (Figure 6), an isoquinoline alkaloid, encapsulated in chitosan and modified chitosan. The berberine-loaded nanoparticles showed improved oral delivery and control release revealing benefits for bones, osteoarthritis [155], intestinal cells protection and activity against *Helicobacter pylori* [156]. A cannabinoid derivative, 1-naphthalenyl [4-(pentyloxy)-1-naphthalenyl] methanone (CB13, Figure 6) was successfully encapsulated in chitosan by Durán-Lobato et al. [157] and the results showed adequate blood compatibility and absence of cytotoxicity in Caco-2 cells indicating that is suitable for oral use. An additional example is geraniol (Figure 6) loaded in chitosan/gum arabic nanoparticles for better pest management [133]. In the study of Oliveira et al. [133], the chitosan/gum arabic nanoparticles loaded with geraniol have been prepared by emulsification, followed by ionic gelation and showed EE > 90%. The chemical structures of the aforementioned natural products belonging in various structural families are shown in Figure 6.

## 4. Chitosan as a Matrix for the Encapsulation of Plant Extracts

Plant extracts are usually obtained from different parts of plants such as leaves, barks, seeds, seed coats, flowers, roots and pulps using various extraction techniques and solvents [158]. Depending on the extraction technique and protocol, the extraction of a certain group of secondary metabolites may be favored. The most widely used extracts are the aqueous, alcoholic and hydroalcoholic. Fractionation is a common practice in order to get rid of undesired compounds [159]. Usually the chemical constituents of plant extracts act synergistically with a number of receptors in the human organism. However, toxic effects can also be synergistic. The “rich fractions” used contain the desirable phytochemicals extracted along with other compounds, usually enhancing the functionality and maximizing the benefits of the product. Reasons for using complex mixtures could be the decrease in activity after isolation, chemical instability of isolated compounds, difficulty in the purification and the possibility to act in multiple targets at once. Extracts require strict quality control and standardization in many parameters regarding safety and efficacy which remains a basic shortcoming since they comprise of many known and unknown components [159].

Despite their tremendous potential, natural products’ use to treat diseases is limited and they have not translated into clinical reality yet. The main reasons for that are their sub-optimal pharmacokinetic and pharmacodynamics properties such as poor water solubility, bioavailability and half-life period and limited dose regimen [160,161]. Their poor stability is another limiting factor and, in some cases, their extremely bitter taste restricts the number of their applications as preservativesin foods [144].

The aforementioned shortcomings are less intense upon encapsulation in nanosized matrices. Solubility, bioavailability of certain active agent, pharmacokinetic profile, toxicity problems, stability and targeting ability are enhanced using nanotechnology [144,160,161]. In addition, nanotechnology is a very useful strategy for cancer therapies, providing a new delivery system of natural products to tumor sites selectively. The name of this blend is ‘nanochemoprevention’ [161]. 

There are numerous studies describing the encapsulation of different extracts in chitosan nanoparticles via, mainly, the ionotropic gelation method with TPP, and their potential applications (Table 1) [122,127,160,161,162,163,164,165]. Beconcini et al. successfully encapsulated Crognola capannile cherry fruit extract (*Prunus avium* L.), in two types of chitosan derivatives. No significant differences were observed between the two types of polymers as both nanosystems showed very high EE ~80%, nanoparticle size around 340 nm, and positive zeta potential around 15 mV. However, biological results indicated high protection of the endothelial cells from oxidative stress which is related to vascular dysfunction that is implied in a number of cardiovascular pathologies, only by the type of chitosan nanoparticles containing protected thiol groups [165,166].

Sanoj Rejinold and coworkers synthesized saponin-loaded chitosan nanoparticles from the ethanolic extract of the plant *Sapinduse marginatus,* following an ionic cross-linking method using TPP. The EE was calculated as 95%, while the nanoparticle size was found to be efficient enough for delivery applications (40–60 nm). The results indicated that the produced CSNPs could act as a therapeutic agent for cancer, showing non-toxic effects to normal cells [163].

Following the same technique, in 2019 Mahmoudi and his group [127] entrapped the hydro-alcoholic extract of seeds of *Physalis alkekengi* in chitosan nanomatrix and optimized the method through response surface methodology. The maximum EE was found to be 95%, while SEM results indicated a uniform nanoparticle size around 167 nm. The antioxidant activity of the produced CSNPs was investigated through two different assays and results revealed improved capacity of the extractand enhanced stability of its bioactive ingredients [127]. Furthermore, CSNPs loaded with distilled aqueous green tea extract (GTE) from the plant *Camellia sinensis* were prepared by Safar and coworkers, leading to nanoparticles of 200–250 nm size and positive zeta potential of 40–50 mV. Synthesized GTE CSNPs were successfully evaluated in removing all the extracellular collagen caused by CCl_4_ in the hepatic fibrosis rat liver [160].

Barrera-Necha et al. [167] encapsulated botanic extracts in CSNPs investigating their antifungical activity, against *Alternaria alternata* and *Colletotrichum gloeosporioides*, for use in coatings or packaging in commodities postharvest. The methanolic extract of nanche (from plant *Byrsonima crassifolia*) loaded on chitosan revealed the most stable suspension with zeta pontial value of -43.8 mV and the lowest particle size (304.2 ± 31.7 nm), compared to ethanolic blueberry extract (from *Vaccinium corymbosum*) loaded on chitosan nanoparticles and to blank nanoparticles. In vitro experimental results indicated the effective antifungal activity of nanche extract CSNPs against *C. gloeosporioides,* while blueberry extract incorporated in CSNPs proved to be weak against *A. alternate* [167].

In another study, it was revealed that loading a blueberry-derived mixture of anthocyanins in CSNPs slowed the breakdown of anthocyanins in simulated gastrointestinal fluid and improved their stability [168]. Furthermore, in the study of Alfaro-Viquez and coworkers, a cranberry proanthocyanidins extract from the plant *Vaccinium macrocarpon* was encapsulated in chitosan, leading to increased stability, as well as molecular adhesion to extra-intestinal pathogenic *Escherichia coli* [164].

In 2016, Yulianti et al. perfomed the ionic gelation method for the encapsulation of *Centella asiatica* ethanolic extract in CSNPs, exhibiting its promising potential as an anti-aging cosmetic [122]. Moreover, Kailaku and and coworkers achieved the development of the optimum formulation of the catechin (gambier) extract, from the plant *Uncaria gambier* Roxb, loaded in CSNPs, with the particle size of 137.6 nm, for its application as antioxidant material [169]. 

Swarnalatha et al. encapsulated an alkaloid extract from *Sphaeranthus amaranthoides* in modified chitosan-alginate nanoparticles and they evaluated their anti-cancer activity in A549 lung cancer cell lines. Results showed that alkaloids act as good apoptotic inducers against tumors [170]. 

In 2018, Ntohogian and his group [5] applied the ionic gelation method in order to prepare CSNPs loaded with natural and ultrafiltrated extracts from saffron and annatto. DLS and SEM results demonstrated that the size of all the produced CSNPs ranged from 150 to 500 nm, while their shape was spherical or irregular. Additionally, physicochemical characteristics of the prepared emulsions were enhanced, whereas their sunscreen protection factor was weak (SPF value from 2.15 to 4.85) [5,171,172].

In the research study of Zou et al., cocoa-procyanidins extract (CPs) from cocoa beans was encapsulated in gelatin-CSNPs, leading to particles of 344.7 nm size, zeta-potential value of 29.8 mV and spherical morphology. The results indicated that the encapsulation of the CPs extract improved its stability, demonstrating good apoptotic effects in human acute leukemia cells [173].

Gaber Ahmed et al. recently reported the encapsulation of phenolic extracts from golden apple and red grape in chitosan using the nanoemulsification-solvent displacement method and Tween 20 as the emulsifier. The antioxidant activity of the nanocapsules was determined using the DPPH radical scavenging assay and the results showed that the nanocapsules enhanced the antioxidant activity of the phenolic extracts [129].

The extract from the rhizome of turmeric containing curcumin (~77%), dimethoxy-curcumin (~17%) and bisdemethoxycurcumin (~3%) (“curcuminoids extract”) has been incorporated in chitosan-gelatin microcapsules of spherical shape. The preparation was succeeded via complex coacervation, using Tween 80 as the emulsifier and formaldehyde as the cross-linking agent. The resulting microcapsules led to increased water solubility of the extract. The release of curcuminoids was faster from the non-crosslinked microcapsules and slower from the cross-linked ones and followed zero order kinetics, which is the desirable release profile for sustained drug release [174].

Two types of nanocarriers, namely chitosan and Soluplus polymeric micelles were used for the encapsulation of the hydroalcoholic extract of *Posidonia oceanica* (L.) Delile. The CSNPs were prepared using the ionic gelation method with TPP. Although both nanosystems improved the aqueous solubility of the extract, only the polymeric micelle system, which possessed higher encapsulation efficiency and better release profile, was able to inhibit cancer cell migration [175].

## 5. Chitosan as a Matrix for the Encapsulation of EOs

EOs are natural, volatile, hydrophobic and concentrated liquids with a pronounced odor isolated from plants. EOs are a rich source of a wide range of bioactive but sensitive chemical compounds. Despite this promising potential their poor aqueous solubility, and their sensitivity to the different environmental conditions such as light, oxygen, chemicals, heat, pressure, pH and moisture during food processing, limit their use in the pure form. As a result, EOs can easily become unstable and lose their biological features. For example, heat exposure of EOs may cause epimerization, oxidation, and degradation of some of their chemical compounds. Their sensitivity can also lead to adverse effects in humans such as hypersensitivity reaction and allergic dermatitis due to the chemical conversion of some constituents after atmospheric exposure [3,119,176,177,178,179,180,181]. Another problem is the high volatility of EOs [116] and one more limitation is their irregular dispersion in food industry use [2].

Plant EOs are generally recognized as safe [3] with negligible side effects and cost effectiveness [2]. They possess a broad spectrum of activities such as antifungal [104], antioxidant and antimicrobial activities [176]. Several EOs exert beneficial effects towards inflammation and cardiovascular disorders [2], urging the scientific community to focus on effective delivery systems for the EOs.

Nanoparticles as a delivery system represent a viable, efficient and promising approach in order to mask EOs’ handicaps and overcome such limitations. Nanoencapsulation is used to protect EOs’ bioactive constituents from evaporation, oxidation and degradation and improve their activities. Moreover, nanoencapsulation can improve thermal stability during processing, storage and transport and reach sustained release profile offering prolonged activity [92] for food and pharmaceutical applications [110,121,176]. Additionally, encapsulation ameliorates water-solubility and bioavailability of lipophilic compounds [103], reduces the toxicity and cost of bioactive compounds due to the reduction in the required quantity [127] and lastly protects flavors for engineered delivery in functional foods [177].

There are many examples of EOs encapsulated in chitosan nanostructures, the majority of which involving the emulsion-ionic gelation technique [2,3,4,103,176,178].

Jamil et al. [113] loaded cardamom EO in chitosan nanocapsules using the ionic gelation method. The encapsulation efficiency was more than 90% and the size was estimated in the range of 50 to 100 nm. The produced nanocapsules effectively controlled in vitro the multidrug resistant *E. coli* and methicillin-resistant *S. aureus* without toxicity to human cells. 

Barzegar et al. [107] prepared thyme EO-loaded chitosan nanocapsules by emulsion-gelation method (EE between 27 and 42%), in order to enhance thyme EO’s thermal stability and antioxidant activity. Sotelo-Boyás et al. [110] studied the release and inhibitory activity against foodborne bacteria of thyme EO and carvacrol, loaded in chitosan nanoparticles and nanocapsules sized 6.4 ± 0.5 nmand 9.1 ± 1.6 nm, respectively, presenting EE 68 and 72%. 

Haider et al. [114] investigated the use of encapsulated krill oil (*Euphausia superba*) in CSNPs (EE between 33 and 59%) using an oil-in-water emulsification and ionic-gelation method for application as a dietary supplement. The loaded CSNPs (80–130 nm) led to the prevention of EO oxidation. 

The study of Ferreira and co-workers [181], on the encapsulation of *S. guianensis* EO in CSNPs chemically crosslinked with glutaraldehyde, showed promising results offering an alternative solution for larvicide control. All the tested chitosan/EO ratios had better larvicidal activity than just the oil without adjuvants. Depending on the chitosan/EO ratios the EO contents in the nanoparticles were estimated between 28 and 58% and the EE was calculated between 85and 87%, respectively. 

The encapsulation of clove EO by CSNPs was performed by Hasheminejad et al. [103] using a two-step process, including formation of an oil-in-water emulsion and ionic gelation of emulsion droplets to improve the antifungal efficacy of the EO. The loaded CSNPs were spherical with size up to 100 nm and the EE calculated between 31 and 46%, depending on the chitosan:EO ratio. The same research group, studied the effect of different coating dispersions such as chitosan, clove EO from the plant *Eugenia caryophyllata* and clove EO loaded in CSNPs prepared according to the previously described method, on the quality of pomegranate arils [104]. This study revealed improved antifungal efficacy of EO-CSNPs, extended aril shelf life for 54 days and maintained high quality compared to uncoated EO [104]. The same two-step preparation method was used by Shetta and co-workers [3], for the encapsulation of peppermint and green tea EO in CSNPs. The EE of peppermint CSNPs and green tea CSNPs were up to 78–82% and 22–81%, respectively. Both CSNPs showed thermal stability that reached 350 °C and an enhanced antibacterial activity against both *S. aureus* and *E. coli*. Their antioxidant activities were also improved compared to the free EO. Hadidi et al. [105], also encapsulated clove CSNPs obtained from hydro-distillation of air-dried clove buds in chitosan nanoparticles applying the oil-in-water emulsification method (using Tween 80 as the emulsifier) followed by TPP-induced ionic gelation. The loaded nanoparticles showed higher antioxidant activity than the free EO and also showed potent antimicrobial activity against *Listeria monocytogenes* and *Staphylococcus Aureus*.

*Coriandrum sativum* EO entrapped in CSNPs by Das et al. [2] was tested for its antifungal, antiaflatoxigenic and antioxidant activities. In this study, the encapsulation of EO in chitosan matrix (the size of encapsulated EO nanoparticle ranging between 57 and 80 nm) was achieved by oil-in-water emulsion technique followed by homogenization of EO in chitosan and TPP solution through ionotropic gelation. The results were very promising even for commercialization of enhanced shelf-life and avoidance of fungal contamination for stored rice [2]. Improved antifungal and antimycotoxin activity against *Fusarium graminearum* were shown by CSNPs with *Cymbopogon martini* EO with a promising use in agricultural and food industries [119]. In this study, Kalagatur et al. [118] prepared spherical EO-CSNPs via emulsification technique with zeta-potential of 39.3–37.2 mV and ranging between a size of 455 and 480 nm. CSNPs with *Cinnamomum zeylanicum* EO were tested for their antimicrobial activity. The EO was encapsulated by the ionic gelation technique into chitosan nanoparticles presenting an average size of 100–190 nm, an EE 2–17% and loading capacity 3–4%. The results showed that nanoparticles decrease both the severity and incidence of infected cucumbers by *Phytophthora drechsleri* extending their shelf life [178].

Another study showed a good antimicrobial activity of thyme oil-loaded CSNPs (6.4 ± 0.5 nm) and CSNCs (9.1 ± 1.6 nm) especially against *Staphylococcus aureus* and *Bacillus cereus*, respectively [110]. Good antibacterial properties against food-borne pathogens were found in the lime EO-loaded CSNPs (6.1 ± 0.4 nm) and the lime EO-loaded CSNCs (6.1 ± 0.6 nm), being higher for the nanoparticles especially against *Shigella dysenteriae* than for the nanocapsules [115]. Two more EOs were tested for their antimicrobial activity, peppermint oil from *Mentha piperita* and green tea oil from *Camelia sinensis* both loaded in CSNPs. The results showed weak antibacterial activities against *Staphylococcus aureus* for the first one and better antibacterial activities against *Staphylococcus aureus* and *Escherichia coli* for the second one [3].

Ashrafi et al. [112] in 2019 encapsulated *Mentha piperita* EO in CSNPs using the sol-gel method with TPP as the linking bridge and tested their ability to inhibit biofilm formation from *Staphylococcus mutans* and protect against bacterial dental mineralization. Indeed, the loaded nanoparticles effectively inhibited the biofilm formation and were found to specifically inhibit some glygosyltransferase genes. The release kinetics of the nanoformulation in hydroalcoholic solution showed that about 50% of the EO was released after 360 h.

Bitter orange oil from the plant *Citrus aurantium* was successfully incorporated in CSNPs (20 and 60 nm) using TPP as cross-linkerand the results from the study revealed improved antioxidant activity and microbial safety as well as higher antioxidant enzymes activity of white button mushroom (*Agaricus bisporus*) [176]. Lemon EO from the plant *Citrus limon* L. was also successfully entrapped in nanocapsules, of average size from 339.3 to 553.3 nm, based on chitosan and modified starch (Hicap) with desirable physicochemical properties and stability for future use in medicine and food industries [177].

The summer savory EO is as well known and studied EO due to its use in food, medical and pharmaceutical industry [179,180]. Feyzioglu et al. [4] encapsulated the summer savory EO from the plant *Satureja hortensis* L. into CSNPs using the ionic gelation method. The EO-CSNPs were obtained at pH levels of 4.5, 6.0 and 10.0, their size ranging from 140.3 to 237.6 nm and their EE evaluated between 35 and 41%. They possessed strong antibacterial activity against *Staphylococcus aureus*, *Listeria monocytogenes* and *Escherichia coli* as well as good antioxidant activity. The results from the entrapped *Siparuna guianensis* EO in chitosan by an oil-in-water emulsion with an EE ranging from 84% to 88%, demonstrate its use as a potential larvicide control alternative against mosquito *Ae*des *aegypti,* a vector of infectious diseases such as yellow fever [181]. Rosemary EO from *Rosmarinus officinalis* encapsulated in CSNPs by Hussein et al. [6] using homogenization technique with an EE up to 80%, showed enhanced stability in thermal processing applications, useful in food and pharmaceutical industry.

A summary of phytochemicals, extracts or essential oils that have been encapsulated in chitosan nanoformulations as well as the bioactivity of the nanosystems is presented in Table 1, Table 2 and Table 3.

## 6. Chitosan-Coated and Modified Chitosan Nanosystems Encapsulating Natural Products

In pharmaceutical nanotechnology, chitosan is not only used as a matrix-forming polymer [182] for the encapsulation of bioactive substances but also as a surface coating material. Drug delivery nanosystems decorated with chitosan are commonly polymeric nanoparticles mainlypoly(lactic-*co*-glycolic acid) (PLGA), poly(lactic acid) (PLA), poly(caprolactone) (PCL) or natural polymers, alginates, oligosaccharides (e.g., CDs [183]) and lipid-based nanoparticles such as liposomes [184], solid lipid nanoparticles (SLNs [185]), nanostructured lipid carriers (NLCs [186]) and metal nanoparticles.

Chitosan-coated nanoparticles (CCNs) have been developed as multipurpose material for a myriad of applications. CCNs present a series of advantages such as a) improved physicochemical properties of the nanoparticles (e.g., water solubility, viscosity) b) enhanced mucoadhesiveness and tissue penetration properties, c) tailored-controlled and prolonged drug release, d) enhanced anti-inflammatory and antimicrobial activity and d) improved drug bioavailability [66].

In particular, coating could modulate several physicochemical properties of nanoparticles, such as their aqueous solubility and viscosity as it has been confirmed by several research studies [187,188]. For instance, Abd-Ellatef et al. [189] reported that chitosan-coated SLNs containing curcumin (Table 4), were characterized by a hydrophilic surface as the lipophilic chains of chitosan fit inside the solid lipid carrier and the -OH groups were oriented outward.

Amine groups of chitosan render the CCNs positively charged presenting, therefore, a higher extent of cellular internalization due to the ionic interactions with the cell membranes. Additionally, owed to their charge CCNs are able to escape from lysosomes in the interior of the cells presenting perinuclear localization. On the other hand, it should be mentioned, that negatively or neutrally charged nanoparticles prefer to co-localize with lysosomes [190]. Moreover, due to this higher extent of internalization it has been investigated that the highly positively charged chitosan enhances the cytotoxicity of various drug-loaded nanoparticles against different types of human cancer cell lines [189,191].

Chitosan-surface-modified nanoparticles seem to increase nasal delivery of compounds such as the natural product resveratrol [192] and simvastatin [193], due to chitosan’s mucoadhesive and penetration-enhancing properties [194,195,196], chitosan through the reversible opening of the tight junctions increases the paracellular transport [193]. Chitosan coating process constitutes also a new strategy which overcomes hurdles such as the limited brain-blood barrier (BBB) permeability presented by several drugs against the central nervous system (CNS) and nullifies the treatment against CNS diseases [192,193]. However, CCNs used through the nose-to-brain route could deliver the drug more effectively to the CNS bypassing the BBB [196]. According to Bahadur et al. [197] nose-to-brain route is more efficient due to the direct connection via the olfactory system.

In general, CCNs provide two diffusional barriers for the release of the encapsulated compound: (a) the internal core (nanoparticle) and (b) the polymeric coating. This double diffusional barrier release is desirable for specific applications and depending on the release conditions could lead to tailormade release profiles. Arafa et al. [198] support that chitosan physical barrier limits the diffusion and surface erosion of the encapsulated compound from the PLGA nanoparticles, which in addition to the limited tendency of chitosan to hydrate when it is in high pH dissolution media, leads to the elimination of burst release effectand drug release retardancy [74]. On the other hand, chitosan coating in acidic conditions seems to increase the release of several drugs such as diclofenac. Thus, pH sensitivity of chitosan in combination with the nature of the drug [199,200] offers pH responsive chitosan nanostructures [201,202,203]. Furthermore, the viscosity of chitosan is a physicochemical property that also affects the release of the drug in the local area [201]. N. Üstündag˘-Okur et al. [202] presented that high viscosity CCNs lead to slower drug release due to the higher viscosity of the gel layerwhich is formed when the nanoparticles and the release medium interface [203].

The enhanced anti-inflammatory activity of CCNs is attributed to the fact that chitosan in slightly acid conditions presents inherent anti-inflammatory, antibacterial and antimicrobial activity enhancing therefore the bioactivity of drug loaded nano-systems [204]. For instance, the positive surface charge of chitosan allows it to interact with negative microbial surfaces, preventing microbial growth or causing increased death rate [205].

Surface chitosan modified nanoparticles could be prepared either by electrostatic deposition (physical absorption) or by chemical modification (chemical binding). Specifically, electrostatic deposition is based on the electrostatic interactions between the positively charged ammonium groups of chitosan in slightly acid conditions with the negatively charged surface of the matrix material [66,206].

**Figure 7:**
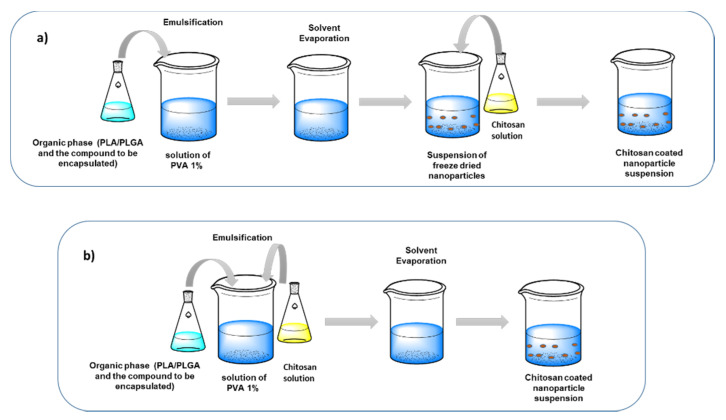
Schematic illustration of different strategies used for the formation of chitosan-coated poly (lactic acid) (PLA)/poly (lactic-*co*-glycolic acid) (PLGA) nanoparticles; formation of chitosan-coated nanoparticles (CCNs) (**a**) using pre-formed negatively charged nanoparticles and (**b**) during the formation of the nanoparticles.

Moreover, electrostatic deposition could be applied for the formation of CCNs either in already formed negatively charged nanoparticles or during the formation of the nanoparticles (Figure 7). Both approaches have been used to prepare coated polymeric nanoparticles, lipid nanoparticles and metal-based nanoparticles as well [183,186,188,189,206,207,208]. Especially, natural products such as curcumin [209] and resveratrol [192] were successfully encapsulated in chitosan-coated nanoformulations where the coating process was accomplished using pre-formed nanoemulsions and lipid microparticles, respectively.

On the other hand, plant-derived bioactive compounds such as forskolin, ferulic acid and quercetin were successfully delivered by CCNs, where coating was achieved during the nanoparticle formation [210]. Preparation of CCNs via electrostatic adhesion while using pre-formed nanoparticles, initially required the dissolution of chitosan in acetic acid aqueous solution. Then, the prepared solution was added to the nanoparticle suspension of defined concentration, and the mixture was stirred and sonicated for specific time [211]. In case of the preparation of the desired CCNs during the encapsulation process, a single emulsion solvent evaporation technique was applied. The aqueous phase containing the AA chitosan solution and the surfactant solution is emulsified with the organic phase, containing the polymer and the drug. The solvent used is evaporated and the CCNs are collected [212,213].

Preparation of CCNs by chemical modification could be achieved for polymer-core nanoparticles containing carboxylate ending groups e.g., PLGA, PLA or PCL using activating and dehydrating agents such as N-hydroxysuccinimide (NHS) and *N*-(3-dimethylaminopropyl)-*N*′-ethylcarbodiimide [214] hydrochloride (EDC·HCl). Briefly, the freeze-dried nanoparticles previously prepared are dispersed in phosphate buffer solution (PBS) of slightly acidic pH (pH 6.0) using sonication. Afterwards, activating and dehydrating agents are added to the buffer solution and thereafter the chitosan solution [215].

The success of the coating process closely depends on factors/parameters such as the concentration of chitosan, [200,214] the concentration of nanoparticle suspension, the DD [216] and MW of chitosan [194,209]. Chitosan that is used for coating material applications could be of various MWs commonly of low and medium MW.Li et al. demonstrated the use of different MWs of chitosan for the preparation of curcumin-loaded nanoemulsions [209]. Low MW chitosan was used for the coating of tobramycin-loaded PLGA nanoparticles leading to a slower drug release compared to uncoated nanoparticles [207]. In the case of nanoliposomes coated with chitosan, it seems that encapsulation efficiency and release kinetics are affected by the molecular weight and the concentration of chitosan [216].

The successful coating process of negatively charged nanoparticles with chitosan is proven by the alteration of the zeta-potential from negative to positive [217]. In addition, differences in size of the coated nanoparticles compared to the uncoated ones have been illustrated.

Matshetshe et al. [183], thoroughly studied the encapsulation of the *Cinnamomum zeylanicum* EO in the system β-CD/chitosan nanosystem presenting that the encapsulation of the EO in the oligosaccharide first differentiated significantly the system. The size of nanoparticles lacking the β-CD ranged from 123.3 to 326.4 nm, their zeta potential was found to be between 24 and 30.5 mV while the encapsulation efficiency (EE) 10–20%, depending on the conditions under which the nanopatricles’ preparation was carried out. However, when the EO had formed with the CD an inclusion complex which was then encapsulated into CSNPs, the size range was between 255 and 415 nm, the zetapotential 20–34 mV and the EE 39–58%.

In the work of Almalik et al. [218], a comparison of chitosan, alginate/chitosan and hyaluronic acid (HA)/CSNPs is conducted. It was found that the non-coated nanoparticles were sized 170 nm, the PDI was 0.3 and the zetapotential was found to be 34 mV. On the other hand, the incorporation of alginate, led to nanoparticles sized 790 nm with significant heterogeneity (PDI = 0.46) and a very strongly negative zetapotential (−72 mV), attributed to the presence of alginate on the exterior surface of the nanoparticle. The coating of CSNPs with HA resulted in nanoparticles of diameter 270 nm, with better uniformity (PDI = 0.22) and zeta potential-32 mV. The study of the three nanosystems’ capacity to absorb 19 proteins with inflammatory-related activity, revealed significant differences between them: none of the proteins tested were absorbed by all nanosystems. Moreover, the latter nanosystem exhibited the lowest immunogenic activity [218,219]. Furthermore, it has been shown that low MW chitosan forms smaller nanoparticles compared to high MW chitosan due to its higher aqueous solubility and shorter polymer chains [194,220].

As already mentioned, there is a variety of matrices that are commonly used with chitosan. Alginic acid, PLGA and HA are linear, hydrophilic polymers that are often used in biomedical and food applications. Due to their anionic nature, they can interact with chitosan electrostatically, forming biocompatible and biodegradable nanoparticles, without the use of other cross-linking agents. However, these polymers could also be the coating of CSNPs, in which case the zeta potential would be reversed, due to the outer surface of the occurring nanosystem. On the other hand, chitosan could also be the coating of alginate or HA nanoparticles therefore the encapsulation process is established depending on the requirements of the application studied. Moreover, the hydrophilic nature of alginate, PLGA and HA in combination with chitosan enhances the solubility of the system [219].

An example of chitosan-coated alginate nanoparticles encapsulating quercetin was presented by Nalini et al. [188]. The nanoparticulate system size ranged between 118 and 254 nm and EE between 76 and 82%, depending on the preparation process followed. In vitro study of the release of quercetin in different pH conditions verifies the higher stability of the formulation in lower pH.

PLGA nanoparticles, although they are suitable carriers for the delivery of a variety of natural and synthetic bioactive molecules, possess a difficulty in presenting passive or active targeted effects, attributed to their surface [206]. Thus, many studies focused on the development of chitosan-coated PLGA nanoparticles which, due to their repeating amine group, render the PLGA surface more easily involved in chemical reactions [221]. For instance, Lima et al. [212], prepared a combined nanosystem of chitosan with PLGA for the encapsulation of ferulic acid. The two polymers were self-assembled, forming spherical nanoparticles with average diameter 242 nm and PDI = 0.20, surface charge 32 mV, and entrapped the 50% of the ferulic acid. The release profile of the drug was studied in conditions that simulated the gastric fluid for 2h followed by 4 h in simulated intestinal fluid. Total release of the ferulic acid was about only 15% and occurred mainly in the first 30 min. At pH 7.4 the release rate was high for the first 8 h but significantly lower after. The study of Hypochlorous acid (HOCl) scavenging assay and cytotoxicity over HeLa and B16-F10 tumor cells lines suggested that the encapsulation may increase the biological effects of the phenol, increasing also the permeability through Caco-2 monolayer and triple co-culture of Caco-2/HT29-MTX/Raji B.

Metal nanoparticles have been also coated with chitosan biopolymer developing new hybrid coated nanomaterials which present improved biological and physicochemical properties [213]. Hybrid chitosaninorganic nanoparticles combine materials with different mechanisms of action and have been innovatively developed as improved antibacterial [222], antimicrobial [223], anticancer [224] and wound healing agents [225].

Chitosan-coated silver nanoparticles (AgNPs) are an exceptional example of hybrid nanosystem developed for the enhancement of the antibacterial activity against pathogenic Gram-negative (e.g., *E. coli*) or Gram-positive bacteria such as *Staphylococcus aureus* [226]. In particular, Senthilkumar et al. in 2019 presented the green synthesis of silver nanoparticles coated with chitosan using leaf extract of *T. portulacifolium* as reducing agent [208]. The obtained spherical shaped nanoparticles have cubic fluorite structure presenting average particles size ranging from 3.2 nm to 44.8 nm as it was indicated by TEM analysis. The antibacterial activity evaluation against *E. coli* and *S. marcescens* organisms was carried out by standard agar well diffusion method revealing that chitosan-coated AgNPspresented satisfactory antibacterial activity at the concentration of 50 μL. Moreover, at the same concentration FESEM analysis illustrated that the hybrid nanosystemexhibited noticeable morphological changes against both pathogenic bacteria. Cell membrane damage is responsible for cell death due to the disruption of coated nanoparticles on microorganisms’ membrane surface [226,227].

Regarding the cytotoxic activity of chitosan-coated inorganic nanoparticles it has been reported by Wu and Zhang that chitosan-coated zinc oxide nanoparticles (ZnO) present cytotoxic effects attributed to the higher internalization of the metal ions (Zn^2+^) in the cancer cells, compared to the uncoated negative charged nanoparticulate. Especially, chitosan-coated zinc oxide nanoparticles prepared by physical absorption after the formation of the ZnO nanoparticles, were evaluated for their cytotoxicity against cervical cell lines. The results demonstrated increased cytotoxicity of the coated NPs while the presumable mechanism of killing effect was the intracellular ROS generation [228]. Moreover, the differences in the cell morphology are indicative of the cell death via apoptosis [223].

Even more elaborate systems can be formed to meet with the application’s requirements. In the work of Song et al. [229], carboxymethyl-β-CD was grafted on chitosan prior to the nanoparticles’ formation. The model protein drug Bovine serum albumin (BSA) was used then encapsulated in the modified chitosan matrix. This encapsulation technique resulted in the EE of the protein between 13 and 77%, depending on the initial loading and similarly sized nanoparticles around 190 nm. The release profile of BSA significantly differentiated when conducted in different pH, simulating the pH gastric fluid, intestinal fluid and simulated colonic fluid. At pH 1.2, only about 18% of the drug was released in three days, while in pH 6.8 the 48% of the drug was released and the pH 7.4 the 70%. The findings suggested that the particular nanosystem could be used for oral drug administration, enhancing the drug’s bioavailability.

Modified chitosan derivatives have been innovatively presented as functional materials due to their exceptional physicochemical properties and biological activities. They overcome the main drawback of chitosan, its poor solubility in water and polar organic solvents [230]. Zu et al. [152], used carboxymethyl chitosan (CMCS) for the nanoencapsulation of resveratrol, proposed for oral administration. This chitosan derivative is water soluble in all the pH range with low-toxicity, enhanced biocompatibility and antimicrobial property [231].

The encapsulation of the natural phenol into spherical nanoparticles, sized 155.3 nm and surface charge of −10.28 mV was succeeded by emulsion cross-linking of COO^−^ group of the CMCS with the Ca^2+^ ion of anhydrous calcium chloride. The EE was calculated to be 45%. The biological effects of resveratrol were amplified by the encapsulation due to a 3.5216-fold increase in its bioavailability, attributed to higher absorption of the molecule within vivotesting in rats.

Campos et al., grafted β-CD on chitosan glycol to form the nanoparticles. The glycol derivative of the polymer due to its high solubility in aqueous solutions facilitates the functionalisation of chitosan. Briefly, the encapsulation process was the following: first, the inclusion complexes (ICs) of the oligosaccharide with carvacrol or linalool were prepared. Then, chitosan was chemically modified with the IC using the crosslinking agents EDC and NHS. Finally, the nanoparticles were formed through ionic gelation. The aim of this study was to increase the solubility of the two volatile compounds as well as their insecticidal activity. The size of the carvacrol-loaded nanoparticles was 175.2 nm and the zetapotential 13.5 mV. The linalool-loaded nanoparticles had a mean diameter of 245.8 nm and zetapotential 17.3 mV. In both systems the EE was higher than 90%. The effectiveness of the two compounds was enhanced with simultaneous decrease in their toxicity. Expanding the study, the two monoterpenes were co-loaded in a similar system. The particles that occurred had mean size (225.9 nm) and good homogeneity (PDI = 0.185) and zeta potential = 19.3 mV. The release study at 25 °C revealed different profiles for each compound.In both profiles, an initial burst release was observed, but the release rate of carvacrol was lower than that of linalool. Therefore, 600 min after the beginning of the release study, 49% of carvacrol was released, while linalool release was 71% after 460 min [232,233].

Chen et al. [187], synthesized the arginine-modified chitosan which was self-assembled with thiolated fucoidan for preparation of multifunctional nanoparticles for the co-encapsulation of curcumin and BSA. The loaded nanoparticles’ size was found to be 147.1 nm and presented significant uniformity (PDI = 0.18), the zeta potential was 24.2 mV and the EE of curcumin 72%. The encapsulation significantly increased the solubility as well as the stability of the phenol in aqueous solutions. The release rate of curcumin was examined in two different conditions: at pH 2.0 simulating the gastric fluid and pH 7.4 simulating the intestinal fluid. It was found that the total release of curcumin at pH 7.4 after 25 days was over 60% while at pH 2.0 it was around 20%. It was confirmed that the combination of the two polymers enhances paracellular and transcellular delivery of hydrophilic and hydrophobic compounds, as well as their intestinal permeability by opening the tight junctions.

Natural products, that constitute a source for the discovery of new drugs for the current pharmacopoeia providing diverse bio-inspired bioactive molecules [230,234], are meeting with growing success regarding their exploitation, in combination with the “smart” materials which could enhance and prolong their bioactivity. As chitosan and its derivatives present a variety of physicochemical properties and inherent biological activities, they could be used for the development of various multifunctional nanomaterials.

It has been proved that glucosamine chitosan derivative increases the aqueous solubility of chitosan [235] and enhances also its antioxidant activities in neutral pH [236]. Driven by this, Braber and coworkers [210] presented in 2018 the microencapsulation of the natural product quercetin in chitosan and its glucosamine derivative via spray drying method. Encapsulation in both cases improved the chemical and biological stability of quercetin and therefore its bioavailability. Interestingly, they presented that the prepared chitosan microcapsules (with size around 2 μm) are not only a delivery system of a natural antioxidant, but a micro-system which combined the antioxidant activity of quercetin and the intrinsic antioxidant properties of chitosan and glucosamine chitosan. The antioxidant activity of the micro-systems was evaluated through their ability to scavenge hydroxyl and superoxide radicals. Furthermore, the release studies of the obtained microcapsules in gastric and intestinal conditions indicated a greater release profile from the glucosamine chitosan microcapsule which is attributed to the higher aqueous solubility of the chitosan derivative.

Hussain et al. [237], presented the preparation of CSNPs co-loaded with the natural product hydroxytyrosol and the synthetic compound hydrocortisone. Synergistic effects of natural bioactive compounds and synthetic molecules expand the potential treatment strategies for various diseases [238]. Thus, hydroxytyrosol and hydrocortisone were co-administered in order to restrict systematic adverse effects of hydrocortisone, providing simultaneously additional antioxidant (hydroxytyrosol) and anti-inflammatory effects for atopic dermatitis treatment. The obtained CSNPs present EE values of 39% and 33% for hydrocortisone and hydroxytyrosol, respectively. Co-loaded nanoparticles enhanced the accumulation of both drugs in the skin layers, improving consequently the drug delivery in the local area. In addition, ex vivo studies revealed that the co-loaded nanosystem reduced hydrocortisone and hydroxytyrosol permeation across the full-thickness dermatomed skin. In vivo clinical results demonstrated that the bifunctional nanosystem-presenting anti-inflammatory and antioxidant activity is efficient for the alleviations of the signs and symptoms of atopic dermatitis.

Chitosan can also be used as the coating of another nanosystem. Song et al. [239] prepared magnetic alginate/CSNPs, loaded with curcumin, to promote the targeted delivery to cancer cells. More specifically, after the preparation of the Fe_3_O_4_ magnetic nanoparticles, an alginate coating was added producing the magnetic alginate nanoparticles (MAPs). Then, the dried MAPs were further coated with chitosan. Chitosan and alginate, being a cationic and an anionic polymer, respectively, interact electrostatically and form nanoparticles. Therefore, in this study, the two polymers created a layer-by-layer self-assembly matrix coating the iron oxide nanoparticles. The polyphenol was entrapped into the polymeric matrix. The size of the particles ranged from 120 to 200 nm, depending on the number of layers of alginate and chitosan added into the system. The zeta potential changed from strongly negative (about −40 mV) when alginate was the outer layer of the system, to positive (about 20 mV) when chitosan was the external layer. The release rate of the curcumin from nanoparticles with up to nine layers was examined at pH 7.4 and 5.6 and was found to be low for all of them. As the layers increased, the curcumin’s release was prolonged. When alginate was the outer layer, the nanoparticulate system was more stable, thus, the release rate was lower. The enhancement of the anticancer effect of curcumin was verified with the use of Human Caucasian Breast Adenocarcinoma cells (MDA-MB-231). The uptake of curcumin was found to be 3 to 6 times higher compared to the uptake of free curcumin, leading to higher cytotoxicity against the cancer cells. On the other hand, the uptake of curcumin from the non-cancer model cells HDF was significantly lower.

Manconi et al. [240,241] encapsulated curcumin into chitosan-coated and hyaluronan-coated liposomes in order to investigate impact of the coating on the pulmonary delivery of the molecule. The chitosan coating resulted in more lamellar spherical nanoparticles, and a slight increase in the size of the particles was observed. Chitosan coating increased the bilayer thickness from 52 to approximately 60 Å, and was attributed to the interaction between the polymer and the polar part of the phospholipids. The nebulization studies reveal the mucoadhesiveness of the chitosan coating and the ability of the nanoparticulate system to reach the deepest part of the respiratory tree.

Miele et al. [242], chemically modified chitosan with OA to form the resveratrol-loaded, amphiphilic chitosan: OA micelles sized 289 nm. Then, the micelles were coated with PLGA slightly decreasing their size (273 nm), but significantly improving their uniformity, the EE of the molecule and its thermal stability. Zetapotential remained highly positive. X-ray diffraction characterization confirmed that resveratrol is dispersed inside both polymeric matrices. The dual encapsulation system exhibited strong cytotoxic activity against both the colonic adenocarcinoma cell line (Caco-2) and the human cervical cancer cell line (HeLa). It was suggested that the amphiphilic nature of the modified chitosan strongly enhanced the interaction of the nanoparticulate system with the cell substrates. However, the PLGA coating of the system further improved the cytotoxic activity of the system, as it offered a more compact structure.

A very interesting application of CSNPs is the incorporation in films, gels or scaffolds, used as immobilization matrices, which adds value to the system. Leena et al. [243] prepared a complex matrix for the nanoencapsulation of the plant derived flavonolignan silibinin. First, the bioactive compound was encapsulated in CSNPs of 215–264 nm diameter and zeta potential 42.5–48.3 mV, depending on the loading. Ionic gelation with TPP was used for the formulation of the particles and the EE was more than 90%.Subsequently, the system was uniformly entrapped in the pores of alginate/gelatin scaffolds. The final particulate system was 120–150 μm and enhanced the osteo-conductive and osteo-inductive properties of silibinin by prolonging its release and thus, increasing its bioavailability, which in the free form is very low due to the hydrophobic nature of the compound. Therefore, the research team proposed the particular system for tissue engineering.

Medina et al. [244], prepared CSNPs loaded with thymol, sized 293.1 nm, with zeta potential = 47.8 mV and EE = 67%. The producednanoparticles were immobilised in an edible chitosan-quinoa protein film. The incorporation of nanoparticles in the film reduced the amount of water that could pass through the film. Therefore, it was proposed for use as active food packaging. To that end, the films prepared were used as an internal coating on plastic containers of fruits and the shelf-life of the fruits was evaluated. The presence of the nanoparticles extended the shelf-life of the food tested and was attributed to the decrease in the water vapor permeability along with the antimicrobial activity, induced by thymol.

Another food packaging appplication was proposed by Lin et al. [245], who encapsulated moringa oil in CSNPs, and then immobilised the nanosystem in gelatin nanofibers. Briefly, CSNps loaded with the moringa oil were prepared with ionic gelation and were then incorporated in gelatin spinning solution to prepare the fibers with the use of an electrospinning apparatus. The encapsulation of the nanoparticles into the fibers decreased the hydrophilicity of the system and the permeability of water vapor. Moreover, the hardness of the gelatin nanofibers increased while the extensibility decreased with the addition of the nanoparticles. The antimicrobial activity of the system against *Listeria monocytogenes* and *Staphylococcus aureus* existing on cheese was verified.

A promising approach for the development of chitosan based antibacterial film has been introduced by Chabala et al. [246]. Specifically, they prepared a hybrid system of chitosan/alginate blend co-loaded with the beneficial Aloe vera gel and the silver nanoparticles (excellent antibacterial activity) in order to develop an active wound dressing with enhanced antimicrobial activity and co-controlled delivery of potent antibacterial agents.The chitosan/alginate matrices were prepared via blending method studying different ratios while the incorporation of the Aloe vera and AgNPsin various proportions was carried out using immersion method.

In vitro release studies illustrated that the release rate of Aloe vera gel is dependent on the proportion of chitosan and alginate in the prepared matrices. For instance, increased proportion of alginate led to a greater number of the available carboxylate ions (-COO^−^) which could be protonedduring the entrapment of Aloe vera gel. Due to the neutralization of carboxylate group in the acid pH of Aloe vera, an insoluble gel was formed in water which thereafter led to the reduction in the release rate [247]. On the other hand, increased chitosan ratio in the polymeric matrices resulted in the increase in protonated amine groups leading therefore to a fast release of Aloe vera gel. The latter is attributed to the higher solubility of amines ions in acidic pH. According to the in vitro release kinetics studies the mechanism of Aloe vera release follows Fickian diffusion.

The antibacterial activity of the films was evaluated against Gram-positive and Gram-negative bacteria namely, *S. aureus* and *P. aeruginosa*, respectively. The results showed a strong relationship between the antibacterial activity and the release of Aloe vera constituents (anthraquinones, acemannan and salicylic acid) and silver nanoparticles as well. The film with a higher chitosan ratio (increased -NH^3+^) and thereafter with a higher Aloe vera gel release in combination with silver nanoparticlespresented the highest antibacterial activity. Therefore, the antibacterial capacity of this system is attributed to the synergistic effect of Aloe vera, AgNPs and the polymeric matrix surface [246].

The chitosan-containing nanosystems in which natural products have been encapsulated as well as their bioactivity are summarized in Table 4.

## 7. Overview and Perspectives

Chitosan-based nanostructures seem to be promising systems for the exploitation of a variety of naturally derived products since the sensitivity of the latter in adverse environments restricts their efficacy and their bioavailability. A wide range of essential oils, plant extracts and pure phytochemicals have been incorporated into chitosan nanoparticles, presenting enhanced biological activities and improved physicochemical properties and stability. Thus, chitosan nanoparticles show expanded applicability in the pharmaceutical, food, agriculture and cosmetic industries. CSNPs delivering naturally occurring compounds have been studied for the development of drug delivery systems for anti-cancer targeting and treatment, for wound healing and as antioxidant, anti-inflammatory and antibacterial agents. The therapeutic potential of CSNPs is expanded via co-delivering natural bioactive compounds and synthetic drugs possessing synergistic effects.

Except for matrix material, chitosan is currently used as a coating material enhancing the properties of a variety of polymeric and lipid-based nanoparticles loaded with natural origin products. Chitosan-coated nanoparticles offer great advantages including among others the enhanced mucoadhesiveness and tissue penetration properties, the tailored, controlled and prolonged drug release and the enhanced anti-inflammatory and antimicrobial activity. Modified chitosan derivatives, obtained by tuning the surface chemistry, have recently gained interest since they present higher aqueous solubility, customized release profile and improved biological activities, leading thereafter to tailor-made systems with distinctive properties. The final customized properties of the chitosan nanoparticles and chitosan-coated nanoparticles are closely related to several parameters such as the molecular weight, viscosity and degree of deacetylation and are defined depending on the desired requirements for the various applications.

In the currently existing literature, chitosan nanoparticles have been incorporated into films, nanofibers gels or scaffolds. Especially, films containing chitosan nanoparticles have extensively gained interest for edible food packaging applications due to their beneficial physicochemical properties and inherent antimicrobial activity.

However, the determination of the in vitro and in vivo toxicity and safety of chitosan-based nanostructures is urgently required. Moreover, current research focuses on the optimization of chitosan nanoparticles preparation, the improvement of their stability and biocompatibility, and thereafter the enhancement of their effectiveness in various applications. Additionally, concerning the chitosan chemically modified derivatives, as a future prospect, green and eco-friendly synthesis routes should be developed.

As a result, the beneficial properties of chitosan and its modified derivatives render them “smart” materials for the development of multifunctional nanomaterials for a wide range of applications.

## Figures and Tables

**Table 1:** Chitosan as a matrix for the encapsulation of natural products.

*Source Name*	Extract	Biological Study	Main Constituents	Chitosan (CS) Characteristics	Preparation Method	Outcome	Ref.
***Centella*** ***asiatica***	*C. asiatica* ethanolic extract	Microculture tetrazolium assay for analysis of the proliferation of normal human dermal fibroblasts (NHDF) and normal human epidermal keratinocytes (NHEK), test on type I and III collagen synthesis using ELISA, immunocytochemistry in combination with ImageJ software for the evaluation of Aquaporin 3 expression	Asiatic acid, madecassic acid, asiaticoside and madecassoside	CS with a deacetylation degree >70%	Ionic gelation	Anti-aging activity by inducing skin cell (fibroblasts and keratinocytes) proliferation and AQP3 expression	[122]
***Physalis*** ***alkekengi***	Hydro-alcoholic extract of seeds of *P. alkekengi*	Non-biological but antioxidant assays: DPPH, FRAP	Physalins, carotenoids, alkaloids, polyphenols, flavonoids	Low MW CS	Ionic gelation using TPP	Improved antioxidant capacity	[126]
***Theobroma cacao***	Golden apple and red grape	DPPH assay			Nanoemulsification-solvent displacement method and Tween as the emulsifier	Enhanced antioxidant activity	[129]
	Cocoa bean procyanidins (CPs) extract	Cell apoptosis with annexin V staining and cytotoxicity assay in the THP-1 cell line	Procyanidin oligomers (from monomer to decamers) and polymers, with polymers being the predominant component	CS (low MW, 75–85% deacetylated)	Preparation of CPs-gelatin-CS nanoparticles	Improved stability and good apoptotic effects at lower concentrations in human acute monocytic leukemia THP-1 cells	[173]
***Camellia*** ***sinensis***	Green Tea Extract (GTE) distilled water extract	Uptake study in HepG2 cells, test on carbon tetrachloride (CCl4)-induced hepatic fibrosis in rats	epicatechin gallate(ECG), epigallocatechin (EGC), epicatechin(EC) and caffeine	Water-soluble, low MW CS obtained from mushroom	Ionic gelation using TPP	Effective in removing all the extracellular collagen caused byCCl4 in the hepatic fibrosis rat liver	[159]
***Allivum*** ***sativum***	Garlic aqueous extract	In vitro drug release			Ionic gelation	High stability and in vitro release for future use in many diseases such as cancer	[162]
***Sapindus*** ***emarginatus***	Sapindus extract with distilled ethanol	Specific cytotoxic assay (MTT) against prostate/oral cancer cells/normal cells	Saponin	Average molecular weight (MW) 20 kDa, degree ofN-deacetylation (75–80%)	Ionic gelation using TPP	Potential therapeutic agent for cancer, inducing dose-dependent cancer cell death with lower toxicity on normal cells	[163]
***Vacciniumma*** ***crocarpon***	Cranberry proanthocyanidins (PAC)	Determinationof the effect on the (extra-intestinal pathogenic *Escherichia coli*) ExPEC invasion of gut epithelial cells in vitro	Flavonolglycosides, anthocyanins, proanthocyanidins, and hydroxycinnamic acids, but use only of proanthocyanidin enriched fraction (PAC)	CS from shrimp shells (deacetylation degree of 92%, MW185 kDa	Ionic gelation	Increased stability and molecular adhesion of PAC to ExPEC	[164]
***Prunus avium L.***	Crognola cherry fruits extract	In vitro test on HUVECs (Human umbilical vein endothelialcells)stressed with H_2_O_2_	Polyphenols	S-protected thiolated derivative		Protection of the endothelial cells from oxidative stress related to vascular dysfunction implied in a number of cardiovascular pathologies.	[165]
***Vaccinium corymbosum***	Blueberry fruit ethanol extract	In vitro antifungal evaluation (sporulation and germination were measured) on *Alternaria alternata* from *Ficuscarica* and *Rosmarinus officinalis*	Flavonoids, phenolic acids, tannins, and anthocyanins	Medium MWCS (deacetylation degree 75–85%)		Weak antifungal activity against *A. alternata* from fig and rosemary	[167]
***Byrsonima*** ***crassifolia***	Nanche leaves methanol extract	In vitro antifungal evaluation (sporulation and germination were measured) on *Colletotrichum gloeosporioides* isolated from *Carica papaya* L. and *Annona muricata* L.	Fatty acids, diterpenes, phenolic compounds and monoterpenes	Medium MW CS (deacetylation degree 75–85%)		Improved control of *C. gloeosporioides* isolated from papaya and soursop leading to synergistic effect	[167]
***Uncaria gambier Roxb.***	Catechin (gambier) extract	No-biological assays, DPPH assay	Higher levels of catechin (42%): catechin acid and catechu tannat acid and small quantity of quercetin	CS (deacetylation degree: 85%)		Good particle surface topography, internal structure of the particles and emulsion stability, good antioxidant activity	[169]
***Sphaeranthus*** ***amaranthoides***	Alkaloid extract		Alkaloids, tannins, saponins, flavonoids, alkaloids, proteins and steroids	CS-alginate nanoparticles		Good apoptotic inducer in vitro, inhibition of the cell growth via induction of apoptosis in A549 cell line.	[170]
***Crocus sativus***	Saffron and ultrafine saffron aqueous extract	In vitro cytotoxicity study measuring the viability of HUVE cells incorporation in sunscreen emulsions (emulsion stability and SPF determination assays)	Crocin-1, crocin-2, crocetin, safranal	CS with high MW (MW: 350,000 g/moL, deacetylation degree >75%, and viscosity 800–2000 cps)	Ionic gelation using TPP	Formed nanoparticles with spherical and irregular shape, and size varied from ~150 to ~500 nm, crystalline dispersion, for sunscreen emulsions: good stability, viscosity, low cytotoxicity.	[171]
***Bixaorellana***	Annatto and ultrafine annatto (UF)	In vitrocytotoxicity study measuring the viability of HUVE cells incorporation in sunscreen emulsions (emulsion stability and SPF determination assays)	Carotenoids, apocarotenoids, sterols, aliphatic compounds, monoterpenes and sesquiterpenes, triterpenoids	CS with high MW (MW: 350,000 g/moL, deacetylation degree >75%, and viscosity 800–2000 cps)	Ionotropic gelation method using TPP	Formed nanoparticles with spherical and irregular shape, and size varied from ~150 to ~500 nm, amorphous dispersion in the case of annatto and UF annatto, for sunscreen emulsions: good stability, viscosity, low cytotoxicity.	[172]
**Rhizome of turmeric**			Curcumin (~77%), demethoxycurcumin (~17%) and bisdemethoxycurcumin (~3%)		Complex coacervation, using Tween 80 as the emulsifier and formaldehyde as the cross-linking agent		[174]
***Posidonia oceanica (L.) Delile.***	Hydroalcoholic extract				Ionic gelation method with TPP	Improvement of the aqueous solubility of the extract	[175]

**Table 2:** Chitosan nanoparticles loaded with essential oils.

**Source/Plant Name**	**Essential Oil**	**Biological Study**	**Main Constituents**	**Chitosan Characteristics**	**Preparation Method**	**Outcome**	**Ref.**
***Coriandrum sativum***	*C. sativum* essential oil (CSEO)	14 different food borne mold swere used for fungitoxic spectrum determination, determination of AFB1 inhibitory efficacy, ABTS•+ assay, TPC determination, phytotoxicity assay	Linalool (65.18%), geranyl acetate (12.06%) and α-pinene (4.76%)	MW = 193,400	Ionic gelation	Efficient broad spectrum antifungal, antiaflatoxigenic and antioxidant agent, inhibitor of methylglyoxal (aflatoxin inducer), inhibitor of AFB1 (aflatoxin B1) secretion	[2]
***Camellia sinensis***	GreenTea oil (GTO)	Agar dilution and colony counting methods against Gram-positive (*S.aureus*) and Gram-negative bacteria (*Escherichia coli*), DPPH assay	Monoterpenes, terpene alcohol, sesquiterpene and phenolic compounds such as flavanones and flavanols	Medium MW CS (84.8% degree of dealkylation)	Emulsification/ionic gelation	High antibacterial activities against *S. aureus* and *E. coli*	[3]
***Mentha piperita***	Peppermint oil	Agar dilution and colony counting methods against Gram-positive (*Staphylococcus aureus* and Gram-negative bacteria (*Escherichia coli)*	Oxygenated terpenoids: menthone and menthol	Medium MW CS (84.8% degree of dealkylation)	Emulsification/ ionic gelation	Weak antibacterial activity against *S. aureus*	[3]
***Rosmarinus officinalis***	Rosemary essential oil	DPPH assay, TPC determination with Folin-Ciocalteu assay	1,8 cineole, camphor, α-terpineol, α-pinene, camphene	Low MW CS	Homogenization	Increased thermal stability	[6]
***Eugenia caryophyllata***	Clove essential oil (CEO)	Pour-plate technique for antifungal assays against *Aspergillus niger* isolated from spoiled pomegranate	Eugenol, phenylpropanoid, eugenyl acetate, monoterpeneester and β-caryophyllene, a sesquiterpene	Medium MW and 75–85% degree of deacetylation	Emulsion-ionic gelation using TPP	Promising natural fungicide with improved efficacy against *Aspergillus niger*	[103]
**Hydrodistillation of air-dried clove buds**	Clove essential oil (CEO)				Oil-in-water emulsification followed by TPP induced ionic gelation	Antioxidant activity and potent antimicrobial activity against *L. monocytogenes* and *S. aureus*	[104]
**Thymus (plant)**	Thyme essential oil (TEO)	Six bacterial strains: *S. aureus*, *L. monocytogenes*, *B. cereus*, *Salmonella typhi*, *Shigella dysenteriae* and *E. coli* tested using agar plate technique	Thymol and carvacrol	Medium MWCS (deacetylation degree75–85%)	Two different procedures for nanoparticles (CSNPs and nanocapsules (CSNCs) preparation	TEO-CSNPs had the highest inhibitory activity against *Staphylococcus aureus* and TEO-CSNCs against *Bacillus cereus*	[110]
***Mentha piperita***			l-Menthol 45.05% L-menthalone 17.53% Menthofuran 8.58%, cis-Carane 8.22%, neo-Menthol 4.33%, 1,8-Cineole 4.26% etc.		Ionic gelation	Loaded nanoparticles effectively inhibited the biofilm formation and were found to specifically inhibit some glygosyltransferase genes	[112]
**Citrus species**	Lime essential oil	Four strains of bacteria: *Staphylococcus aureus*, *Listeria monocytogenes-Shigella dysenteriae*, and *Escherichia coli*, were used astest microorganisms in agar plate	Limonene and otherterpenes	Medium MW CS (deacetylation degree75–85%)	Nanoparticles preparation: nanoprecipitation, oil-in-water emulsion followed by ionic gelation and nano- encapsulation preparation: oxidative degradation of medium MW CS using the solvent displacement technique	Synergistic effect in the antibacterial activity against testedpathogens, greater for the nanoparticles compared to the nanocapsules for *S. aureus, L. monocytogenes*, *S. dysenteriae*, and *E. coli* with the highest antibacterial activity being against *S. dysenteriae*	[115]
***Cymbopogon martinii***	*C. martinii* essential oil (CMEO)	Antifungal activity determined by the microwell dilution method on mycotoxigenic, *F. graminearum*, determination of intracellular ROS, lipid peroxidation and ergosterol	Geraniol, geranial, geranyl propionate, geranyl acetone, geranyl acetate, a-phellandrene, and linalool	High purity CS: 99%degree of deacetylation, and MW of 100 kDa		Enhanced antifungal and antimycotoxin activity against *F. graminearum*	[118]
***Citrus aurantium***	Bitter orange essential oil	Inoculated potato dextrose agar (PDA) media for yeast and mold determination and inoculated plate count agar (PCA) for aerobic mesophilic and psychrophilic bacteria determination, determination of glutathione reductase (GR) and peroxidase (POD) activity	Monoterpenes, limone, pinene, synephrine alkaloids, limonoids, phytosterols, flavonoids including hesperidin, naringin and nobiletin	MediumMW CS, 190–310 KDa, viscosity: 200–800 cP, degree of deacetylation: 75–85%	Ionic gelation using TPP	Improved microbial safety and antioxidant enzymes activity (glutathione reductase (GR) and ascorbate peroxidase (APX)) of white button mushroom (*Agaricusbisporus*)	[176]
***Citrus limon* L.**	Lemon essential oil		22 compounds of which limone in the largest proportion, C-pinene, J-terpinene, p-cymene	Low MW with 75–85% DD and modified starch (Hi-cap)	Freeze-drying	Highest encapsulation efficiency and zeta potential with prolonged release value and improved stability	[177]
***Cinnamomum*** ***zeylanicum***	*C. zeylanicum* essential oil	Antifungal assays performed with the pour-plate method	Cinnamaldehyde, benzaldehyde, (E)-cinnamyl acetate, limonene and eugenol	Medium MW with DD 75–85%	Ionic gelation	Reduction in severity and incidence of infectedcucumbers by *Phytophthora drechsleri* and enhancement of cucumber shelf life	[178]
***Satureja hortensis* L.**	Summer savory essential oil	DPPH assay and antibacterial assay against *E.coli*, *L. monocytogenes*, *S. aureus*	Carvacrol, γ-terpinene and p-cymene	CS from crab shells, 85% deacylated	Emulsion and ionic gelation using TPP	Strong antibacterial activity against *Staphylococcus aureus, Listeria monocytogenes* and *Escherichia coli* and antioxidant activity	[179,180]
***Siparuna*** ***guianensis***	*Siparuna guianensis* essential oil	Bioassay for determination of toxic activity against *Ae*des *aegypti* larvae	Monoterpene *β*-, myrcene, sesquiterpene epicurzerenone, Germacrene D, γ-elemene, non-terpene acyclic ketone 2-undecanone	Viscosity-average MW CS with deacetylation degree 76.5%		Potential larvicide control against mosquito *Ae*des *aegypti* (vector ofinfectious diseases such as yellow fever, dengue, zika, and chikungunya)	[181]

**Table 3:** Chitosan nanoparticles loaded with purified phytochemicals.

Compound Name	Category	Plant Source	Biological Activity of the Phytochemical	Biological Study	Chitosan Characteristics	Preparation Method	Outcome	Ref.
**Baicalein and Quercetin (separately tested)**	Flavone/ Flavonoids/ polyphenols	Onions, many fruits, or in herbs	AQS (anti-quorum sensing) and antibiofilm activities of pure and nanoencapsulated compounds against the bioengineered *E. coli* Top10 biosensor	Stability test, in vitro release assay in the M9 bacterial growth medium, bacterial assays with *E. coli* Top10 biosensor QS assay, antibiofilm assay, cell viability assay, Mammalian cell (MDCK-C7) line cytotoxicity test using MTT assay	MW∼115,000 g/mol and DD∼42%	Preparation method of nanocapsules	Anti-quorum sensing activity against *E. coli* Top 10 and inhibition of biofilm formation	[98]
**Kaempferol**	Flavonol/flavonoids/polyphenols		Anti-inflammatory, anticancer and antioxidant activities	Modulation of QS (quorum sensing) mediated by AI (autoinducers) in model bioassay test systems, QS inhibition against *C. violaceum* CV026 with disc diffusion assay and quantitative determination of violacein inhibition, DPPH assay, FRAP, in vitro release and stability studies	75–85% DD, low MW	Anionic gelation method using TPP	QS (based anti-biofilm) inhibitory against C.violaceum CV026, for effective antimicrobial chemotherapy, good stability	[99]
**Ferulic acid**	Hydroxy- cinnamic acid/ polyphenols		Antibiofilm potential against *C. albicans*	Biocompatibility on hek-293 cell lines by MTT assay, Fesem and fluorescent microscopy, *c. Albicans* biofilm formation test with XTT assay and scanning electron microscopy	Medium MW (190e310 kDa) with 75–85% DD	Ionic gelation	Effective, safe and powerful antifungal (antibiofilm activity against *C. albicans)* agent	[101]
**Ferulic acid**	Hydroxy- Cinnamic acid/ polyphenols	Various cereals, plants and fruits	Antioxidant and anticancer activities, antimicrobial, anti-inflammatory, cholesterol-lowering activities, thrombosis and atherosclerosis prevention, photoprotectiveactivity [132] against diabetes and neurological disorders, antimicrobial, and hepatoprotective activities, and protective effects against the UV, reduction in triglycerides and cholesterol [136]	In vitro antiproliferative potential against ME-180 human cervical cancer cell lines, cytocompatibility evaluation on HEK-293 cells (MTTassay and FESEM analysis)	Low MW (85% DD)	Ionic gelation	Potential therapeutic agent against cancer cells (ME-180 cell lines) proliferation due to apoptotic induction, enhanced cytocompatibility and solubility	[141,146]
**Ferulic acid**	Hydroxy- cinnamic acid/ polyphenols		Anti-diabetic effect due to its antioxidant capacity	In vitrorelease profile, in vivo pharmacokinetic study (in Wistar albino rats), anti-diabetic studies: Oral Glucose Tolerance Test (OGTT) on wistar albino rats, biochemical studies:blood glucose levels, lipid profile and plasma insulin estimation in rat by ELISA kit, histo- pathological study on pancreas of scarified rats	medium MW (190-310 kDa)	Ionic gelation using TPP	Extended plasma retention time, maximum plasma concentration and/or bioavailability and attenuation of the diabetes-associated symptoms	[146]
**Geraniol**	Monoterpene alcohol	Coming from flowers and tissues of many herbs and essential oils (ninde, rose, palmarosa, citronella EO)	Antimicrobial, antioxidant, anti-inflammatory, and antitumor, repellent activity	Photostability and release assays in vitro at different temperatures, biological effects were investigated in whitefly (*Bemisiatabaci*).	CS/gum arabic nanoparticles, CS MW: 27 kDa; degree of deacetylation: 75−85%	Emulsification followed by ionic gelation	Good colloidal properties, improved stability from UV radiation, decreased degradation rates, significant attraction activity against whitefly with potential use in pest management	[133]
**Ellagic acid (EA)**	Hydroxy- Benzoic acid/ polyphenols	(generally) pomegranates, raspberries, strawberries, pecans, blackberries, several vegetables	Antioxidant, anti-proliferative, wound healing properties, coagulation promotion of blood.EA-CS-NPs activities: inhibition of the proliferation of glioblastoma, proliferation of melanoma cells and colorectal cancer cells, against oral cancer cell lines and able to promote apoptosis and DNA fragmentation	Blood clotting time analysis (WBCT) by the Lee-White method and the clot retraction, time (CRT) on rat blood, blood retraction time analysis	CS 85% deacetylated, 140 kDa	Ionic gelation using TPP	Synergism for anti- hemorrhagic activity, efficient promoting blood coagulation factor	[134]
**Curcumin**	Hydroxy- cinnamic acids/ polyphenols	*Curcuma longa*	Excellent antioxidantand antidiabetic properties	In vitro amylase inhibitory activity assay, in vivo antidiabetic assay intissues of rats	DD 78%, MW: 94 kDa; and viscosity = 3 m^2^/s	Ionic gelation using TPP and CS-alginate complex	More effective the CS-alginate- curcumin complex than CS-curcumin, significant reductions in hyperglycemia	[135]
**Curcumin**	Hydroxy- cinnamic acids/ polyphenols	*Curcumalonga*	Anti-cancer and anti-inflammatory properties, anti-bacterial, anti-parasitic and anti-malaria, antioxidant, metal chelating effects in metal toxicity	Evaluation of therapeutic efficacy in arsenic-induced toxic Wistar rats for 4 weeks with many assays	MW 400 kDa		Antioxidant and metal-chelating properties, stable detoxifyingagent for arsenic poisoning, neuroprotective efficacy	[140]
**Curcumin**	Hydroxy- cinnamic acids/ polyphenols	*Curcuma longa*	Antioxidant, anti-inflammatory, anticarcinogenic/ antitumor, and antimicrobial properties	In vitro cytotoxicity assay on HeLa cells (human cervical cancer cell line),in vitrorelease studies	CS: MW ~50 kDa, 90% deacetylated	Tripolymeric composite of alginate (ALG), CS and pluronic. Preparation method: ionotropic pre-gelation followed by polycationic cross-linking	Suitable size distribution, drug encapsulation efficiency, and drug release kinetics in delivery of hydrophobic drugs	[147]
**Curcumin**	Hydroxy- cinnamic acids /polyphenols	*Curcuma longa*	Anticancer properties against breast cancer	Assay for intracellular uptake of curcumin and cell viability with MTT assay on breast cancer cell lines MCF-7 (Her2-) and MDA-MB-453(Her2+) and in vitro curcumin release	Silk fibroin (SF) and CS polymers, SFCS nanoparticles	Devised capillary-microdot technique	Weaker efficacy of SFCS nanoparticles againstbreast cancer cells (and potential for in vivo breast tumor treatment) than SF-curcumin nanoparticles (showed the highest curcumin entrapment, release, intracellular uptake and highest biological activity)	[148]
**Curcumin**	Hydroxy- cinnamic acids/ polyphenols		Blood lipid-lowering, anticoagulant, antioxidant, anticancer activities, various clinical applications	(1) Cytotoxicity and uptake by tumor cells	Deacetylation degree of CS: 95%, viscosity: 100–200 mPas	Ionic cross linking of folate- modified aminated CS (FA-AmCS-TPP) using TPP	(1) Slow and controlled release of curcumin at pH 7.4 (2) Suitable NPs to carry fat-soluble drugs (3) Possible good tumor-targeting effect Potent injectable agents	[149]
**Curcumin**	Hydroxy- cinnamic acids/ polyphenols	*Curcuma longa*	Antioxidant, anti-inflammatory, antibacterial, anticancer activities particularly against colorectal cancer	(1) Ex vivo mucoadhesion study (2) In vitro effect of mucoadhesive interaction between the nanoparticles and colorectal cancer cells.	Low MW CS (75–85% deacetylated)	Ionic gelation using TPP	Better anticancer activity of NPs against colorectal cancer, improved cellular uptake compared to free curcumin	[150]
**(-)- Epigallo-** **catechin-** **3-gallate (EGCG)**	Flavan-3-ols/ flavonoids/ polyphenols	*Camelia sinensis*	Excellent potential in treating/preventing many cancers including prostate cancer	In vivo antitumor efficacy assay on 22Rν1 tumor xenografts in athymic nude mice, PSA (prostate specific antigen) estimation by ELISA, immunohistochemical analysis, inhibition of cell proliferation markers and inhibition of angiogenesis markers assays in mice	water-soluble CS	Preparation in aqueous conditions using TPP	Inhibition of the growth of prostate cancer cells and secreted prostate-specific antigen levels	[136]
**(-)-epigallo-** **Catechin gallate (EGCG)**	Flavan-3-ols/ flavonoids/ polyphenols	Green tea	Generally antioxidant, anti-viral, anti-inflammatory, cardioprotective, neuro-protective and anti-cancer effect	Determination of the stability in the GIT (stomach and jejunum) and plasma exposure in mice			Enhanced oral delivery, plasma exposure, and therapeutic applicationin many diseases	[137]
**Chlorogenic acid (CGA)**	Hydroxy- cinnamic acid/ polyphenols	Generally apples, pears, berries, plum, vegetableslike sweet potato, lettuce, spinach, coffee beans, tea etc.	Anti-obese, anti-inflammatory, neuroprotective, anti-diabetic, antioxidant, anti-cancerous, radio protective, neuroprotectiveproperties, and also for treating Alzheimer’s disease, inhibit oxidation of LDL and thereforeprotect against cardiovascular diseases	In vitroantioxidant assay with ABTS, in vivo pharmacokinetics on wistar male rats	Low MW DD 86.6%	Ionic gelation using TPP	Controlled release profile, preserved antioxidant activity, increased bioavailability	[138]
**Lupulone** **And** **xanthohumol**	Beta-bitter acid and chalcones/ polyphenols respectively	Extract dried hopflowers of *Humulus lupulus* L.	Antimicrobial and antioxidant tests against a Gram-positive (*Staphylococcus aureus*) and Gram-negative bacterium (*Pseudomonas aeruginosa*)		First type of used CS: heterogeneous and of high molar weight, obtained by chemical deacetylation/ partial depolymerization of chitin, Second used CS: obtained through an enzymatic process	Ionotropic gelation method using TPP	Activity against several Gram-positive (*S. Aureus)*, Gram-negative *(P. Aeruginosa*) and *Candida* strains, good stability	[141]
**Naringenin**	Flavanone/ flavonoids/ polyphenols		Anti-inflammatory agent, significant antitumor effects with low toxicity	Antioxidant assays (nitrate scavenging, DPPH, hydroxyl radical scavenging assay), cell cytotoxicity in lung cancer cells by MTT		Ionic gelation using TPP	Significant antioxidant and anticancer (against A549 lung cancer cells) activity	[142]
**Rosmarinic acid**	Hydroxycinnamic acids/polyphenols	*Salvia officinalis* (sage) and *Saturejamontana* extracts and many more	Treatment of vasoproliferative retinopathies, anti-angiogenic activity to retinal neovascularization a mouse model of retinopathy with no retinal toxicity, antioxidant	Mucoadhesion proprieties evaluation by mucin interaction method, cell viabilityon ARPE-19 and HCE-T cell lines and cytotoxicity (using chorioallantoic membrane), permeability studies in cells, transepithelial electrical resistance	low MW (≈50 kDa) with DD 86%,	Ionic gelation using TPP	Efficient drug delivery systems for ocular application in oxidative eye conditions	[143]
**Baicalin**	Flavonoid	Glucoronide of baicalein (Chinese herbal medicine)	Among others, anti-inflammatory, antihypertensive, antifungal, antioxidant, neuroprotective	In vivo biodistribution	CS oligosaccharide lactate (CL, Mn = 5000, deacetylation degree > 90%)	Ionic gelation using TPP	Ιncrease local bioavailability of therapeutic agents in the liver	[151]
**Resveratrol**	Stilbene/polyphenol	Mainly derived from *Polygonum cuspidatum*, grapes and peanuts	Antitumor, antioxidative, anti-bacterial, anti-inflammatory effects, providesprotection against cardiovascular and hepatic diseasesand participates in immune regulation	In vitro DPPH assay, in vitro release assay, in vivo bioavailability studies in Sprague- Dawley (SD) rats	Carboxymethyl CS MW = 14.2 × 10^4^ Da	Emulsion cross-linking method	Increased absorption, prolonged duration of action and increased relative bioavailability	[152]
**Genistein**	Isoflavonoid phytoestrogen		Antioxidant and neuroprotective activity	Ex vivo permeation studies on nasal mucosa, in vitro cytotoxicity studies	Chitoclear^®^ 1360, MW 35 kDa, 96% deacetylated	Ionic gelation with sodium hexametaphosphate as cross-linker	Enhanced genistein penetration through the nasal mucosa	[153]
**Eugenol**	Phenol		Antimicrobial and antioxidant properties	DPPH method	DD 0.95 and MW of ∼760 kDa	Ionic gelation of an oil-in-water emulsion using TPP	Potential antioxidants for various thermal processing applications	[154]
**Berberine (chloride)**	Isoquinoline alkaloid	From Rhizoma *Coptidis* (Huanglian in Chinese)	Osteoarthritis (OA) treatment	Histological analysis, TUNEL staining assay, quantitative real-time polymerase chain reaction, Western blot, and immunohistochemical methods on male Sprague- Dawley rats analyses of caspase-3, Bcl-2 and Bax expressions, in vitro release assay	Mw = 9.0 × 10^5^, DD 90%	Ionic cross-linking method using sodium TPP	Effective anti- 439 apoptosis activity in the rat OA model, potential therapeutic agent for OA	[155]
**Berberine**	Isoquinoline alkaloid	*Berberis vulgaris*, *Berberis aristata*, *Berberis petiolaris*, *Berberisaquifolium*, *Berberis asiatica*, *Berberis thunbergii*, *Coptisteeta*, *Coptischinensis*, *Hydrastis canadensis*, *Phellodendronamurense* and *Caulis mahoniae*	Anti-viral, anti-microbial, anti-diarrhea, anti-inflammatoryand anti-tumor, anti-diabetic, glycolysis stimulator and mitochondrial functioninhibitor, improved lipid and glucose metabolism, for heart failure, cardiac arrhythmia and hypertension		(1) Nano-hydroxyapatite/CS (n-HA/CS) (2) Fucose-CS/heparin nanoparticles (3) CS and fucoidan-taurine (FD-Tau) conjugates (4) CSNPs		(1) Treating bone defects (2) Activity against *Helicobacter pylori* (3) Inhibition of the redistribution of tight junction (TJ) ZO-1 protein and improved intestinal epithelial TJ disruption (4) Protective against osteoarthritis	[156]
**1-naphthalenyl** **[4 -(pentyloxy)-1-naphthalenyl]methanone (CB13)**	Cannabinoid derivative		Analgesic in chronic pain with less penetration into brain	In vitro drug release, human blood compatibility test, uptake assay on THP1 cells and MTT assay on human colon adenocarcinoma cells, Caco-2 cells(ECACC)	low MW 67,000 g/mol, 75–85% deacetylated,	Polymeric poly (lactic-co-glycolic) acid (PLGA) and lipid nanoparticles (LNPs) surfaces have been modified with CS	Adequate blood compatibility and absence of cytotoxicity, good oral carriers for CB13	[157]

**Table 4:** Chitosan-coated and modified chitosan nanosystems encapsulating Natural Products.

Encapsulated Product	Coating	Modification	Chitosan Characteristics	Outcome	Ref.
Curcumin	Curcumin-loaded SLNs with chitosan coating			Down-regulation of P-glycoprotein expression and rescue doxorubicin efficacy against resistant triple negative breast cancer (TNBC) tumors	[189]
Paliperidone	Paliperidone-loaded PCL nanoparticles with chitosan coating		MW: 100–300 kDa	Minimization of stabilizer induced cytotoxicity, cytokine secretion, and oxidative stress response	[190]
Resveratrol	Resveratrol-loaded Lipid microparticles with chitosan coating		Medium MW; 190–310 kDa; Viscosity 200–800 cP, 1 wt. % in 1% acetic acid, 25 °C, DD 75–85%	Enhancement of the targeting of resveratrol to the brain via nasal administration	[192]
Butyric acid	Chitosan-coated liposomes loaded with butyric acid			Increased cytotoxic activity and important anti-inflammatory effects by inhibiting production of cytokines with a central role in liver cell survival.	[204]
Silver	Chitosan–silver nanoparticles		Low MW; DD 75–85%	Chitosan hybrid silver nanoparticles with antimicrobial potency	[208]
Curcumin	Chitosan-coated nanoemulsion		High MW: 190,000–310,000; DD: 85% Medium MW: 30,000; DD: 89.2% Low MW: 3000; DD: 90%	Preparation of a promising delivery system to promote the applications of curcumin in functional food and beverage system	[209]
Quercetin	Chitosan and GACh as coating material for the microencapsulation of Quercetin	Modified chitosan with glucosamine by Maillard reaction (GACh)	Μedium MW (583 kDa) DD 78%	Enhancement of the bioavailability and antioxidant properties of Quercetin	[210]
Amphotericin B and doxorubicin	Chitosan-coated PLGA nanoparticles			Lower cytotoxicity against toward macrophages and active against leishmania in vitro	[211]
Ferulic acid	Chitosan-coated PLGA nanoparticles		Medium MW; DD 75–85%	Promising carriers for oral delivery of Ferulic acid	[212]
Forskolin	Chitosan-coated PLGA nanoparticles		MW: around 110 kDa; DD 96%; viscosity 15cp	Excellent vehicle for forskolin in ocular delivery	[213]
Bovine serum proteins	HA coating of CSNPs and alginate coating of CSNPs		DD > 60% mol, from white mushrooms	Different surface chemistry HA–CSNPs less immunogenic HA–CSNPs adsorb anti-inflammatory proteins Alginate–CSNPs adsorb proinflammatory	[218]
-	HA coating of CSNPs and Alginate coating of CSNPs		DD > 60% mol, from white mushrooms	Optimum cryoprotectant and its concentration for the stability of nanoparticles during freeze-drying process	[219]
-	-		Medium MW and high MW	The size and zeta potential of the particles affect the cytotoxicity	[220]
Curcumin	Nanoliposomes prepared from salmon purified phospholipid coated with chitosan		From shrimp shells, practical grade DD up to 75%	Increase in the dispersion stability Improvement of mechanical stability	[221]
Forskolin	Chitosan–PLGA nanoparticles prepared with emulsion-sonication process		MW: around 110 kDa; DD 96%; viscosity 15cp	Prolonged retention increased effectiveness in reducing the intraocular pressure Ocular tolerance confirmed ex-vivo and in vivo	[213]
Bovine serum albumin	Carboxymethyl-β-CD grafted on chitosan		MW: 4.6 × 104 DD approximately 90–95%	Prolongation of release profiles in simulated intestinal fluid and simulated colonic fluid	[229]
Carvacrol or linalool	Chitosan glycol functionalized with β-CD ICs	Chitosan glycol	Chitosan glycol (≥60% titration), LMW Chitosan	- Repellency and acaricidal effect oviposition activity against *Tetranychusurticae*	[232]
Carvacrol and linalool	Chitosan glycol functionalized with β-CD ICs chitosan nanoparticles crosslinked with gum arabic	Chitosan glycol	Chitosan glycol (≥60% titration; degree of polymerization ≥400)	Decreased toxicity 1. Insecticidal activity against *Helicover paarmigera* and *Tetranychusurticae*	[233]
Curcumin	Consecutive coatings of Fe_3_O_4_ magnetic nanoparticles with PLGA and CS layers			Enhanced anticancer effect - 3–6 fold increase in the uptake of curcumin from cancer cells Lower uptake of curcumin from the non-cancer model cells HDF	[239]
Resveratrol	Chitosan: Oleic acid micelles coated with PLGA	Chitosan oleate	Low MW; DD 80%	1. Strong cytotoxic activity against both the colonic adenocarcinoma and the human cervical cancer cell lines	[242]
Silibinin	Chitosan nanoparticles incorporated in Alginate/Gelatin scaffolds		Low MW; DD 75–85%	Prolongation of release profile Increase in bioavailability 2. osteo-conductive and osteo-inductive properties	[243]
Thymol	Chitosan nanoparticles immobilised in edible chitosan-quinoa protein films		Medium WC; DD 88.5% Low WC; viscosity reduction (ηsp/c) = 203 (mL/g), viscosity average molar mass MW = 269 kDa DD 78.3%	Food packaging extended the shelf-life of the food tested	[244]
Moringa oil	Chitosan nanoparticles immobilised in gelatin nanofibers		85% deacylated	Decreased the hydrophilicity of the system and the permeability of water vapor antimicrobial activity against *Listeria monocytogenes* and *Staphylococcus aureus*	[245]
